# Molecular Determinants of the Human Retinal Pigment Epithelium Cell Fate and Potential Pharmacogenomic Targets for Precision Medicine

**DOI:** 10.3390/ijms26125817

**Published:** 2025-06-17

**Authors:** Cristina Zibetti

**Affiliations:** Independent Researcher, Caravaggio, 24043 Bergamo, Italy; zibettic@gmail.com

**Keywords:** biological sciences, stem cells reprogramming, health sciences, molecular medicine, epigenetics, precision medicine, pharmacogenomics, biotechnology, neuroscience, visual system, complex diseases, United Nations sustainable development goals 5, 8

## Abstract

Age-related macular degeneration (AMD) is a common cause of blindness worldwide, and it is projected to affect several million individuals by 2040. The human retinal pigment epithelium (hRPE) degenerates in dry AMD, prompting the need to develop stem cell therapies to replace the lost tissue by autologous transplantation and restore the visual function. Nevertheless, the molecular factors behind the hRPE cell fate determination have not been elucidated. Here we identify all molecular determinants of the hRPE cell fate identity by comprehensive and unbiased screening of predicted pioneer factors in the human genome: such TFs mediate coordinated transitions in chromatin accessibility and transcriptional outcome along three major stages of the hRPE genesis. Furthermore, we compile a complete census of all transcription factor-specific binding sites by footprinting analysis of the human epigenome along the RPE developmental trajectory. Gene regulatory networks were found to be involved in cellular responses to glucose and hypoxia, RPE nitrosative stress, type II epithelial-to-mesenchymal transition (EMT), and type III tumorigenic EMT, providing routes for therapeutic intervention on pleiotropic targets dysregulated in AMD, diabetic retinopathy, and cancer progression. Genome editing technologies may leverage this repository to devise functional screenings of regulatory elements and pharmacogenomic therapies in complex diseases, paving the way for strategies in precision medicine.

## 1. Introduction

Age-related macular degeneration (AMD) is one of the most common, clinically relevant ocular diseases worldwide, with a global prevalence of 8.7%, an age of onset varying from 45 to 85 years, and a disease burden projection estimated to affect 288 million individuals in western countries by 2040 [1].

AMD causes irreversible visual impairment in people over 60 [2] with polygenic, multi-factorial etiology and an estimated heritability of 48–71%; it is characterized by the early appearance of drusen, pigmentary anomalies of the retinal pigment epithelium (RPE), and progressive photoreceptor dysfunction affecting the macular region. The prevalence of AMD is likely to rise over the next decades in the Western world due to an increased life expectancy.

Thirty-four AMD genetic risk loci were first identified in a large-scale GWAS analysis [3], impacting the complement pathway and inflammation, the innate immune system, HDL transport, and the extracellular matrix organization, and dozens of novel ones have been emerging from meta-analysis in recent years. Nonetheless, most are ascribed to non-coding regions, and they only explain a subset of AMD cases, suggesting that the interplay between epigenetic dysregulation of gene expression, haplotype, and environmental factors such as cigarette smoke, dietary habits, immune dysfunction, and obesity may contribute to the variable penetrance of the susceptibility loci in the pathogenesis of the disease. Single nucleotide polymorphisms (SNPs) can directly impact the relative abundance of a gene transcript, which may serve as a quantitative trait (eQTL). Simultaneous profiling of genetic variation by whole-genome sequencing and gene expression in large-scale cohorts has enabled an agnostic identification of de novo, rare variants and eQTLs, hence stratification of risk by disease-trait associations in intergenic regulatory regions beyond mere GWAS and polygenic risk score. Yet, partial penetrance and cumulative effects of co-occurring SNPs limit the applicability of corrective gene therapies. In spite of a multitude of risk loci, the rationale remains to identify converging downstream effects and mediators so as to suppress or modulate aberrant signaling pathways that are evoked in degenerative conditions and rescue physiological gene expression.

Understanding the key pathological mechanisms in AMD and its epigenetic drivers is, therefore, essential to devise targeted therapies.

The RPE is thought to drive the onset of AMD, with differential chromatin accessibility impacting gene expression first, involving inflammatory pathways, metabolism, homeostasis, and transcription factor recruitment [4]. Differential DNA methylation affects a proto-oncogene involved in TGF beta signaling (SKI) and transcription-dependent DNA repair mechanisms (GTF2H4) [5]; microRNA deregulation has been reported in the macular RPE-choroid [6].

Hypoxia, inflammation, and hyperglycemia, as well as alterations of the RPE tight junctions involved in the permeability of the outer retina-blood barrier, may lead to chronic oxidative damage. Furthermore, a metabolic shift from mitochondrial respiration to glycolysis and cholesterol metabolism leads to complement-mediated mitochondrial fragmentation and apolipoprotein phase separation. Disruption of the RPE circadian clock, dysfunctional rates of photoreceptor outer segment clearance by autophagy or phagocytosis, or impaired lysosomal activity with consequent misfolded protein accumulation also contribute to the RPE dysfunction [7]. As a result, the nucleation and deposition of drusen and hyperreflective foci accompany the pathogenesis of AMD and diabetic choroidopathy [8]. While drugs that promote cholesterol efflux and lipid clearance have shown promising results [9], nanoscale exosomes, nanospheres, and adeno-associated viruses are being developed for the palliative delivery, synthesis, and secretion of active pharmacological ingredients.

Degeneration of the RPE accounts for 90% of all AMD cases, making it an elective target for patient-specific iPSC reprogramming. Ex vivo stem cell tissue derivatives are being implemented for autologous transplantation in the subretinal space to replace the lost tissue and rescue the visual function.

Ex vivo models may serve as a platform to recapitulate the genesis of the human RPE, to mimic the RPE dysfunction in a given isogenic background, screen drugs, and devise genome-editing tools in precision medicine. Nevertheless, the epigenetic determinants and related regulatory elements behind the human RPE cell fate specification and maintenance have remained, hitherto, unexplored.

Here we identify all active transcription factor (TF) binding sites and molecular signatures involved in the human retinal pigment epithelium cell fate determination by transcriptional, open chromatin profiling and comprehensive footprinting analysis of reprogrammed human induced pluripotent stem cells, leveraging a triphasic workflow for clinical-grade autologous RPE [10], which is in use in preclinical studies and ongoing clinical trials.

Through a compendium of TF binding signatures and paired variations in chromatin accessibility and gene expression, we predict human pioneer and regulatory transcription factors that prime all relevant genes for transcriptional activation and repression across three stages of hiPSC-derived RPE differentiation and related regulatory elements with pharmacogenomic potential for treatment of AMD and RPE degenerative conditions.

This work identifies many possible interventional targets in order to modulate the complement system and the inflammatory response of the RPE, inhibit epithelial-mesenchymal transition-related signaling pathways, promote mitochondrial integrity, increase lipoprotein expression and clearance, or leverage protective, hormetic responses to oxidative stress. CRISPRi and CRISPRa tools combined with improved delivery systems to the posterior segment of the eye may as well leverage this epigenetic blueprint to transiently induce, repress, or fine-tune cis-regulatory elements of gene expression in vivo for the homeostatic restoration of the RPE.

## 2. Results

**Figure 1:**
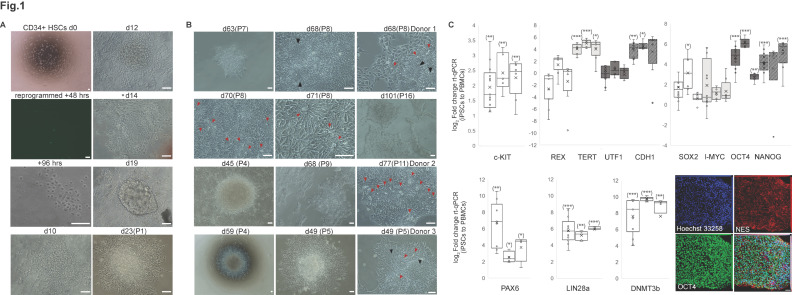
**Phenotyping of hiPSCs.** (**A**) Time-course imaging of reprogrammed HSC CD34+ cells showing pre-iPSC colonies emerging during the first three weeks. Trophoblast-looking structures detectable for some clones (*). Images acquired in brightfield (4×, 10×, 20×, 40×) (e.g., D63 P7) and phase contrast (10, 20×, 40×) (e.g., d101, P16) with an inverted phase contrast Nikon TS100. Scale bar is 50 μm. (**B**) Pre-iPSCs in naïve state form rosettes (black arrowheads) between passages 4 and 7 (P4–P7). The lumen (red arrowheads) emerges afterwards, until passage 12 (P7–P12) towards a primed iPSC state. (**C**) Phenotypic characterization of presumptive hiPSCs by qRT-PCR and immunocytochemistry, with analysis for canonical stem cell markers. Expression fold change in iPSC-derivative cell lines from three donors across passages (one bar per donor) (0–4 months) relative to control (peripheral blood mononuclear cells prior to purification, 72 h before reprogramming) featuring mRNA expression for c-KIT, REX, TERT, UTF1, CDH1, SOX2, l-MYC, OCT4, NANOG, PAX6, Lin28A, and DNMT3b (GAPDH, housekeeping gene). Box-whiskers features log2 fold change (median in horizontal bars, mean as a cross) in hiPSCs relative to PBMC controls. Fold change is 2^(−∆∆Ct)^. Bilateral t-student test (hiPSCs vs. PBMC): |Stat-t| ≥ T α/2 on ∆∆Ct without error propagation. c-KIT (don1, df = 13, *p*-value = 0.0032; don2, df = 6, *p*-value = 0.0026; don3, df = 8, *p*-value = 0.0018), TERT (don1, df = 7, *p*-value = 0.0005 including pre-iPSCs clones; don2, df = 6, *p*-value = 5.73E-05; don3, df = 8, *p*-value = 0.015). CDH1 (pre-iPSCs colonies excluded; don1, df = 9, *p*-value = 0.006; don2, df = 6, *p*-value= 0.0004). SOX2 (don2, df = 8, *p*-value = 0.045); OCT4 (don1, df = 15, *p*-value = 3.39 × 10^−5^; don2, df = 8, *p*-value = 0.0001; don3, df = 10, *p*-value = 0.0044); NANOG (don1, df = 15, *p*-value = 0.0003; don3, df= 10, *p*-value = 0.0006). PAX6 before passage 6 (don1, df = 9, *p*-value = 0.009; don2, df = 4, *p*-value = 0.0189; don3, df = 4, *p*-value = 0.043). LIN28A (don1, df = 15, *p*-value = 0.0002; don2, df = 8, *p*-value = 0.0046; don3, df = 10, *p*-value = 0.0003), DNMT3 (don1, df = 14, *p*-value = 3.69E-05; don2, df = 8, *p*-value = 2.43E-06; don3, df= 10, *p*-value = 0.0023). * applies if *p*-value < 0.05; ** if *p*-value < 0.01; *** if *p*-value < 0.001. Representative image of early neuro-ectodermal progenitors at 3 months from iPSCs derivation is displayed. Cells are stained for NESTIN and OCT-4. Nuclei are counterstained with Hoechst 33258. Confocal image displayed on maximum intensity projection (Zeiss LSM880). Scale bar is 40 μm.

Reprogrammed iPSC clones from three healthy donors were monitored over time following hematopoietic CD34+ cell purification, phenotyping, and expansion (Supplementary Figures S1 and S2). The derivative cell lines were validated by expression of pluripotency markers (Figure 1A–C), along with oncogene exome analysis of hiPSCs vs. donor PBMCs (Table S1).

Overall, canonical stem cell markers such as *SOX2* (sex determining region Y-box 2), *OCT4* (octamer-binding transcription factor 4, also known as POU domain, class 5, transcription factor 1), and *NANOG* (homeobox Transcription Factor Nanog) were robustly expressed across passages in all the cell lines obtained from three donors and retained at later stages, at three months in culture (Figure 1C and Figure S2). *LIN28A* displayed the most pronounced and sustained induction across passages among all stem cell markers, compared to the PBMC controls. *TERT* (telomerase reverse transcriptase), *CDH-1* (e-cadherin), and *DNMT3b* (DNA cytosine-5-methyltransferase 3b) also displayed sustained enrichment across passages in all donor-derived cell lines (Figure 1C), consistent with previous reports.

**Figure 2:**
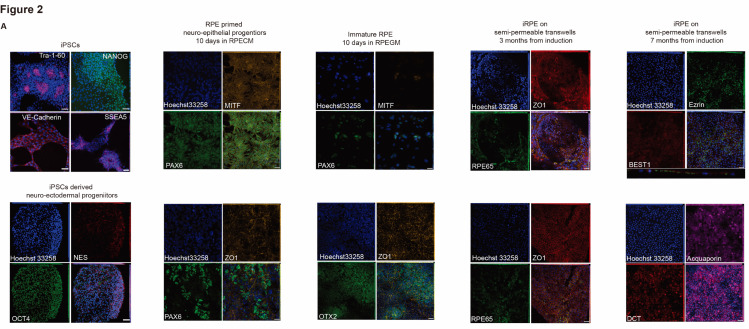

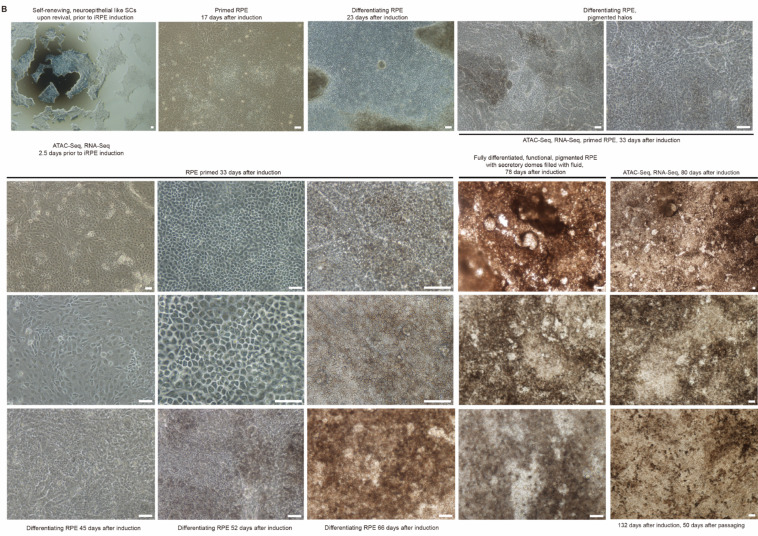

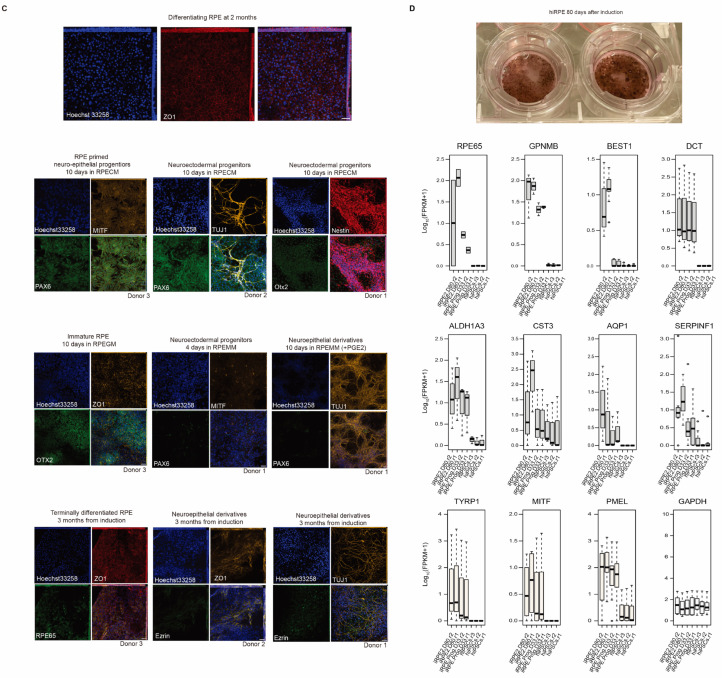
**Generation of hiPSC-iRPE**. (**A**) iPSC cells express markers of stemness and neuroepithelial progenitors. iPSCs rosettes were identified by immunostaining for Tra-1–60, NANOG, VE-cadherin, and SSEA-5 between the second and third months of expansion (maximum intensity projection, Zeiss LSM880. Scale bar is 40 μm), passaged until exhaustion of episomal reprogramming vectors until P10, and retained for iRPE differentiation (see methods). Self-renewing, neuroepithelial-like stem cells were stained with Nestin and Oct4 and counterstained with Hoechst 33258 on their third month of iPSC expansion prior to iRPE induction. The emergence of RPE-primed neuroectoderm, differentiating RPE and induced RPE tissue patches over semi-permeable transwells was monitored over a period of 7 months from RPE induction. (**B**) Stratified differentiative niches and pigmented halos emerge during iRPE genesis. Differentiative niches were monitored over time by brightfield microscopy (4×, 10×, 20×, 40×) and phase contrast (10×, 20×, 40×), leading to fully pigmented, stereotypically cuboidal RPE beehive monolayers after two months. Images acquired with Nikon TS100. Scale bar is 50 μm. Induced iPSCs transition fromconcentric colonies to a layer of disorganized yet evenly distributed cells across the well and, within a month from neuro-epithelial induction, they acquire a cuboidal, globular or fusiform morphology, characteristic of presumptive RPE. hiPSCs cells are interspersed with differentiative niches that are constrained by fibrotic, concentric structures. Differentiated RPE can be retained upon subsequent but limited passages before de-differentiation or phenotypic EMT switch occurs (see methods). (**C**) Bipotent, neuroepithelial progenitors give rise to presumptive RPE, following a hiPSCs mesenchymal-epithelial transition and priming, and neurons. Comparison of three different healthy donor-derived hiPSCs indicates differences in the propensity to generate RPE. hiPSCs derived from three healthy donors. Neuro-ectodermal progenitors were stained after 10 days of exposure to RPECM, 4 days in RPEMM, 10 days in RPEMM (+PGE2), and at three months from induction leading to RPE-primed neuro-epithelial progenitors, immature RPE, and terminally differentiated RPE or alternate neuro-epithelial derivatives. (**D**) Representative screenshot of functionally tested, fully differentiated hiRPE tissue samples (D80) subject to RNA-seq. FPKMs are averaged across transcripts for any given gene. RPE progenitor markers include MITF, PMEL17, and TYRP1. Differentiated RPE markers include RPE65, DCT, BEST1, GPNMB, ALDH1A3, SERPINF1, AQP1, and CST3. FPKM values for transcript ENST00000262340 (RPE65) are 101.67 and 72.7 for terminally differentiated RPE, 3.5 and 1.7 for RPE progenitors, and undetectable in hIPSCs samples.

**Figure 3:**
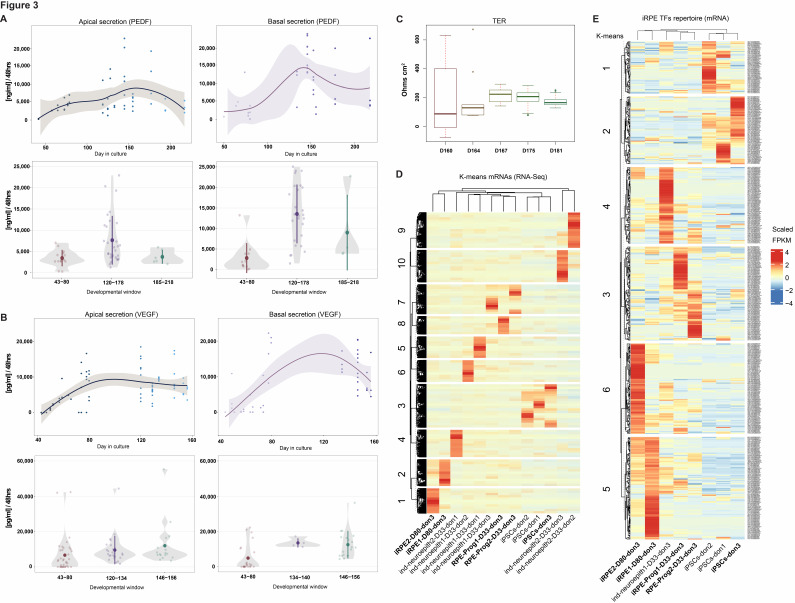
**Functional characterization of hiPSCs-iRPE**. (**A**) Polarized PEDF. Concentration [ng/mL]/48 h was estimated based on points of maximum growth across dilutions. Upper plots display 0.95 confidence interval bands (grey), where the line represents best fit regression line using loess. Apical and basal secretion of PEDF was monitored over the course of seven months from induction. The first temporal window defines RPE cell fate determination, where PEDF secretion is polarized towards the apical compartment. Maximum pigmentation is achieved by day 80 in culture, with fully formed cuboidal, beehive-like stereotypical morphology, eumelanin cells, and secretory domes filled with fluids (point of ATAC-Seq and stage-matched RNA-seq collection). Once passaged, cells are monitored again at d120 for polarized secretion, progressively re-establish tight junctions, cuboidal morphology, and pigmentation. PEDF secretion starts to polarize towards the basal compartment. By the 2nd passage, RPE cells have completely exhausted their proliferative potential and display disorganized monolayers. RPE cells start displaying signs of senescence, altered attachment properties onto the membrane, and depigmentation between the 5th and 6th month in culture. PEDF is primarily secreted towards the apical compartment before full differentiation of RPE is achieved (D80). (**B**) Polarized VEGF secretion. Apical and basal secretion of VEGF was monitored over the course of seven months from induction [pg/mL]/48 h. During the first two months of induction, VEGF polarized secretion occurs predominantly in the apical compartment. One week before maximum melanogenesis is achieved and pigmentation is complete (d80, processing time for ATAC and RNA-Seq), polarized secretion of VEGF becomes predominantly basal. Cells were passaged on D80. (**C**) Transepithelial resistance (TER). TER was measured in mature, differentiated RPE cells (Ohms by cm^2^). (**D**) K-means clustering of all mRNAs across samples exhibiting differences in the propensity to differentiate towards RPE. Assessment of optimal number of k-clusters for k-means clustering of coding mRNAs was carried out by the elbow method. (**E**) iRPE TFs repertoire (mRNAs). K-means clustering of mRNAs encoding TFs was performed. Complex heatmaps display all 564 unique, non-redundant TF IDs and associated mRNA transcripts (highest detected) along RPE differentiation, from iPSCs to RPE progenitors to fully differentiated RPE. Assessment of optimal number of k-clusters for k-means clustering of coding mRNAs was carried out by the elbow method (Figure S5).

Neuro-ectodermal progenitors immuno-stained at 3 months from iPSC derivation were positive for NESTIN and OCT-4 markers (Figure 1C, right panel). Neuro-ectodermal markers such as *OTX2*, *RAX*, *LHX2*, and *MEIS1* were also screened (Supplementary Figure S3), along with mesodermal markers (*NCAM1*, *TBX6*, *HAND1*, *PDGFRa*, *CXCR4*, and *HOPX*) and endodermal markers (*SOX17*, *GATA4*, *FOXA2*, *SMAD3*, *NODAL*, and *cKIT*). The RPE-primed neuroectoderm could be detected after 10 days in RPE commitment medium (RPECM) (PAX6, MITF, and OTX2). The neuroepithelial morphology was already detectable after two weeks, although the presumptive RPE tissue was still morphologically disorganized (OTX2, ZO1) (Figure 2A,B). PAX6 and MITF expression progressively decreased as previously reported [10], indicating a shift towards an immature RPE phenotype. The presumptive RPE started displaying signs of pigmentation across stratified differentiative niches and pigmented halos within one month from neuro-epithelial induction (Figure 2B). Differentiative niches (Figure 2A, RPE65; ZO1) of fusiform, globular, and cuboidal cells were scattered across pigmented halos visible by brightfield microscopy (Figure 2B, D33), eventually merging together to form a homogeneously distributed, monolayered epithelium across the trans-wells. Mature RPE65-positive induced RPE cells (iRPE) established ZO1 expression and a cuboidal morphology upon passaging onto semi-permeable transwells (Figure 2A–C). The RPE progenitors transitioned from a pheomelanin stage to a fully pigmented eumelanin stage during the first two months of iRPE induction (Figure 2B, D66). The resulting tissues display a stereotypically cuboidal, hexagonal morphology visible by bright field and phase contrast, organized in a beehive-like, functional monolayer that expresses mRNAs for canonical RPE markers (Figure 2D), secretes polarized PEDF (Figure 3A and Figure S4) and VEGF (Figure 3B and Figure S4), and has transepithelial resistance (Figure 3C). Hence, the RPE melanogenesis and differentiation were complete by the third month of induction, with secretory domes filled with fluid (Figure 2B,D, D78–D80). The iRPE started to display signs of altered permeability and senescence after seven months from induction (Figure 2A, aquaporin, DCT), depigmentation and epithelial thinning, alteration of tight junctions, and morphology.

### 2.1. Bipotent, Neuroepithelial Progenitors Give Rise to Presumptive RPE Progenitors, Following hiPSCs Mesenchymal-Epithelial Transition to a Primed State, and Neurons

Comparison of three different healthy donor-derived hiPSCs indicates differences in the propensity to generate RPE and alternate neuro-ectodermal derivatives (Figure 2C and Figure 3D). Neuro-ectodermal progenitors from all three donors were consistently positive for stemness and neuroepithelial markers (NESTIN, OTX2, PAX6, TUJ1), leading to neuroepithelial derivatives that progress to an RPE-primed state following hiPSCs mesenchymal-epithelial transition, where the early induction of MITF was predictive of RPE cell fate specification.

The batches that failed to undertake an RPE-cell fate and, instead, displayed neuronal features by phenotypic analysis (Figure 2C and Figure 3D) had limited expression of *MITF*, *TYR*, *TRP1* (*TYRP1*), *TBX2*, and *NFkB1* mRNAs compared to RPE progenitors (Figure 3D,E). Instead, they displayed neuronal transcripts such as *NOTCH1*, *NES*, and *TUBB3*, with *LMX1A*, *ASCL1*, *PITX3*, *MSANDT3*, and *MSX1*, known to be expressed in dopaminergic neurons [11].

Differences in the RPE generative capacity were seen across hiPSC samples that failed to induce *LHX2* mRNA expression at 1 month (D33) from the start of the differentiation procedure. LHX2 is expressed in neuroepithelial, bipotent progenitors that give rise to segregated, neuroretina-like and RPE-like domains in hiPSC-derived optic cups and organoids [12]; it regulates chromatin accessibility and predicted pioneer factors in murine retinal progenitor cells, orchestrating cell fate specification programs [13], and it is required for the maintenance of RPE gene expression in a mouse model in vivo (*Dct-Cre*; *Lhx2^loxP/loxP^*) and in human embryonic stem cells derived RPE [14]. Sustained expression of *PAX6*, *MITF*, *OTX2*, *SOX9*, and *SIX3* mRNAs was also found in RPE progenitors and differentiated RPE, consistent with previous evidence indicating that these factors are able to induce trans-differentiation of human fibroblasts into human RPE-like cells when ectopically expressed [15].

### 2.2. Transcription Factors Footprinting for the Agnostic Identification of Human RPE Master Regulators

We screened open chromatin for enriched gene ontologies along three stages of the iRPE genesis from pluripotency, through RPE-committed precursors, to terminally differentiated RPE (Table S2). Then, we set out to identify all predicted pioneer and regulatory factors that modulate chromatin accessibility by priming it for gene expression or reducing its accessibility to the transcriptional apparatus, resulting in gene repression, and we identified biological functions regulated by such TFs (Table S3).

Finally, we empirically detected all TF binding signatures in the human RPE epigenome by comprehensive, genome-wide ATAC-Seq footprinting analysis of any factor exhibiting detectable levels of stage-matched gene expression by RNA-Seq (Figure 3E and Figure S5).

**Figure 4:**
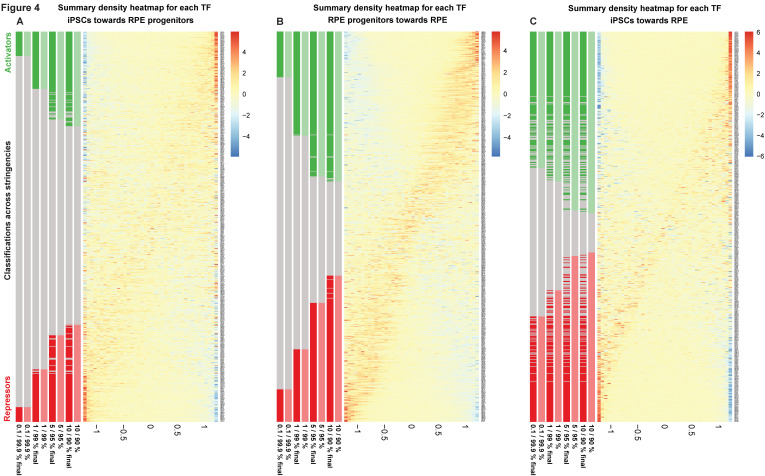
**Summary density heatmap for each TF and classification of activators and repressors across stringencies sorted by the median Pearson correlation**. TF expression levels (x) are correlated with chromatin accessibility at TF-specific target sites compared to non-targeted peaks (peaks bound by unrelated TFs) (y) across different stringencies. 0.1 reflects 0.001% stringency (Figure S9). If the correlation between TF expression level and chromatin accessibility at TF-bound target peaks is more positive than the correlation computed in the background distribution at non-targeted peaks, then the TF is classified as putative activator; if it is more negative, then it is classified as putative repressor; and if the correlation is indistinguishable from that in the background, the TF is classified as undetermined. Briefly, accumulation of red shades represents higher density calls and defines the regulatory activity of any given TF as activator, repressor, or undetermined, whereas blue-shifted values indicate depletion in activity. Thus, expressed activators are clustered on the top right part of the heatmap, enriched for positive Pearson’s correlation values, and represented in green (**left side**, classification by stringency). Repressors are clustered on the bottom left part of the heatmap, represented in red (**left side**, classification by stringency), and are enriched at negative Pearson’s correlation values. TFs with low or conflicting signals placed in the middle, classified as undetermined. Expressed activators result in TFBS (FTPs) within open chromatin at target genes and related gene expression. In such cases, TF footprints are mostly distributed within open chromatin, where the accessibility extends over 100 bp from motif centers, but they are also detectable in closed chromatin with lower read coverage and mostly limited to 10 bp from motif centers. When the expression of TFs is reduced, so is the chromatin accessibility and gene expression of the related target genes, and unused PWMs now populate most targeted chromatin regions, besides few residual footprints in open chromatin regions. Expressed repressors, instead, result into positive correlation with TF binding activity and gene repression of target genes. FTPs are detectable in closed chromatin, with lower reads coverage limited to 10 bp from motif centers, besides unused PWMs.

**Figure 5:**
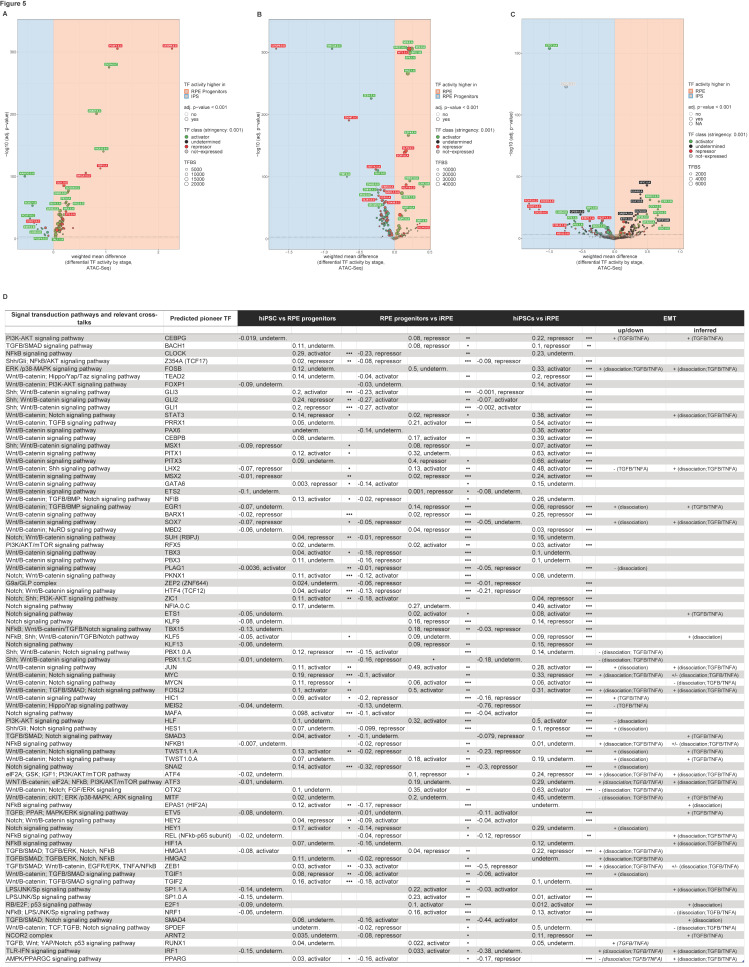
**Differential TF activity and pioneering function in human RPE differentiation.** (**A**–**C**) Volcano plots display differential TF activity (weighted mean difference) between the two experimental conditions reported (*x*-axis) and corresponding significance (*y*-axis) adjusted by multiple hypothesis correction and −log10 transformed. Each dot is a TF. A subset of representative TFs is displayed. Besides a regulatory classification, the comprehensive list of all TFs identified is reported in *.summary.xls, where the sign of the weighted mean difference indicates the directionality of the TF activity, hence the developmental stage, between the two being compared, the TF exerts its highest regulatory function (activity) in, whether as activator, repressor, or undefined. Hence, for positive values of weighted mean difference, the TF is preferentially active in the latter condition being mentioned (i.e., RPE in the IPCSvsRPE.summary.xls file), whereas negative weighted mean difference reflects higher regulatory activity the former (IPSCs). The significance threshold is the dotted line. TFBS is the number of predicted binding sites overlapping ATAC-seq peaks and upon which the differential binding activity (weighted mean difference) is based. Red stands for repressor, and green stands for activator. The identified TFs were subject to Gene Ontology enrichment (see method 4.5) by Fisher’s exact test and corrected by FDR (Table S3). (**D**) Predicted pioneers with detectable expression by RNA-Seq, sorted by cellular phase. Signal transduction pathways are reported. For each TF, the weighted mean difference is reported, along with regulatory directionality in any given cellular phase and statistical significance of the relevant classification; one dot stands for q0.05, two dots for q0.01, and three dots for q0.001. In the “inferred” tab, predicted pioneers are either inferred targets of a given TF from EMT IPA pathway analysis or inferred upregulators/downregulators of EMT molecules.

Additionally, we screened coordinately accessible modules of open chromatin along the human iRPE development for enriched novel oligonucleotides and known TF-related motifs (Supplementary Figures S6 and S7) and identified footprinted genetic targets within overrepresented gene ontologies (Table S4). Last, we classify all TFs as putative activators, repressors, or undetermined across stringencies (Figure 4 and Figure S8) [16] and plot TF differential activity by pairwise comparison across RPE stages of differentiation (Figure 5 and Figure S9). Representative IGV custom tracks featuring progressive changes in chromatin accessibility and corresponding gene expression at target genes *RPE65*, *PAX6*, and *LIN28* along the iRPE genesis are displayed (Supplementary Figure S6C,F,I).

Here, we detect LHX2 repressor activity on shared targets in hiPSCs compared to iRPE progenitors, including neuroectodermal factors (*FOXD3*, *FOXN3*, *HMGN2*, *SALL4)* and genes related to DNA methylation and cell cycle progression (*CCND1*, *MYC*, *KIF20B*, *MTOR*, *TLE4*, *BRD4*, *TET1*, *TET3*), consistent with previous work in murine retinal progenitor cells suggesting that LHX2 may preserve pluripotency by preventing precocious differentiation of subsequent and alternate cell fates [17,18]. Later on, during the transition between human RPE precursors to differentiated RPE, LHX2 activates, instead, gene expression involved in the cellular response to cortisol, Wnt-protein binding, protein kinase C binding, cell cycle checkpoint function, the maintenance of mitotic sister chromatid cohesion, pigmentation, and negative regulation of axon guidance. Ultimately, LHX2 primes chromatin opening for transcription in the transition between human iPSCs to terminally differentiated RPE, consistent with previous findings showing that LHX2 coordinates variations in chromatin accessibility through co-regulatory modules of gene expression and epistatic regulation of cis-regulatory loci of target genes [19].

TFs footprinting analysis on chromatin regions revealed LHX2 binding sites on putative regulatory regions of *PAX6* and *MITF* (Table S4 Footprints that are referenced across the text are reported with TSS distance and related chromosomal locations at predicted distal regulatory and proximal promoter regions). *MITF* regulatory regions were also bound by other predicted pioneering factors, such as OTX2, OVOL1, PAX6, and TWIST1 (Table S4). Furthermore, LHX2 was footprinted on putative regulatory regions of *OTX2*, *POU4F2* (*BRN3B*), *PTF1A*, *RORB*, *VSX2* (also associated with *ABCD4*), *NEUROD1*, and *NR2E1*. LHX2 footprints were also identified in proximal promoter regions for *SOX5* (−456, TSS), *PITX1* (−574, TSS), *NRL* (−148, TSS), *HDAC9* (−443, TSS), and *OPA3* (−269, TSS), among its many identified targets. Another relevant target was *WIF1* (−652, TSS), a key inhibitor of the WNT/β-catenin signaling pathway involved in the RPE cell fate specification and an onco-suppressor gene that is epigenetically silenced by DNA hypermethylation in colorectal cancer and hepatocellular carcinoma [20,21]. We identified additional iRPE developmental repressors that are known to be involved in the maintenance of pluripotency, in the control of neural progenitor cell proliferation and timing of neurogenesis, such as ARID3A, GLIS2, GLIS3, HES1, ZNF322, and ZNF335.

MITF, a master regulator of the RPE differentiation and homeostasis, was footprinted on *VEGFB* proximal promoter (−2, TSS) and *VEGFA*, with major clinical relevance in neovascular AMD and proliferative diabetic retinopathy (Table S4). MITF was also found to regulate *OTX2* and proximal promoters of *LIN28A* (−356, TSS), *NEUROD1* (+69, TSS), as well as sirtuin genes such as *SIRT1* (−56, TSS) and *SIRT4*, which represent potential therapeutic targets for several age-related diseases [8].

OTX2, a key player in the RPE cell fate determination, was footprinted on *MITF*, *BHLHE40,* and the proximal promoter of *CRX* (−64, +4,841, TSS). OTX2 was also footprinted on *CRB1* regulatory regions, as well as *FOS*, *RB1*, *TGFB2*, *TGFB1*, *NEUROG1* promoter (+2042, −7993, −28550 TSS), and *NEUROG2* (−4460, TSS) (Table S4). Among its many identified targets, we found the metabolic sensor and stress response apoptotic mediator *TRIB3* (−351, TSS) involved in the regulation of the AKT/mTOR axis with relevance in autophagy, lysosome biogenesis, energy metabolism, protein and lipid synthesis, cytoskeleton organization, and cell survival. 

Notably, we could also identify OTX2 binding sites on *TRPM1* (+95286, +38, −140426, TSS), a transient receptor potential melastatin ion channel expressed in the RPE, the mutations of which have been reported in association with pigmentation defects and retinal disorders [22]. A *TRPM1* expression quantitative trait locus (eQTL) in AMD was recently shown to be affected by LHX2-OTX2 co-regulatory binding on its promoter region [14]. eQTLs impacting the RPE gene expression arise from non-coding genetic variants in AMD [23] and may result from alterations in chromatin accessibility [8], TF recruitment, and differential DNA methylation on relevant promoter regions [24].

### 2.3. Predicted Pioneer Factors in the Transition from Human-Induced Pluripotent Stem Cells Towards a Retinal Pigment Epithelium Cell Fate

Empirical detection of all TF binding signatures in the human RPE epigenome, computation of chromatin accessibility, and stage-matched variations of gene expression in paired developmental stages (*.all.TF_vs_peak_distribution.xls; *.all.peaks.xls) led us to predict TF activators and repressors (Figure 4 and Figure S10), their differential activity along the human RPE developmental trajectory (Figure 5 and Figure S11, *.all.summary.xls) and related ontologies (Table S3).

Representative pioneer factors predicted as activators, repressors, or TFs with undetermined function (i.e., potentially bivalent) are displayed in the volcano plot (Figure 5A–C), along with their differential binding activity across iRPE stages by pairwise comparison. Some TFs activate gene expression consistently across iRPE developmental stages, inducing chromatin opening (Figure 5D) (PRRX1, CEBPB, JUN, FOSL2, RFX5, PKNX1, MAFA, ETS1, HLF), whereas others act as constitutive repressors across stages (HES1, CEBPG, BACH1, Z354A i.e., TCF17, EGR1, BARX1, SOX7, MBD2, SUH i.e., RBPJ, ZEP2 i.e., ZNF644, KLF9, TBX15, KLF13). Staged repressors repress gene expression preferentially in RPE progenitors compared to the iRPE (NFKB1, PBX3), in the iRPE compared to RPE progenitors (ETS2), or in hIPSCs compared to the iRPE (MEIS2). Staged activators like FOSB, NFIA, PAX6, and PITX1, instead, induce gene expression in iRPE compared to hiPSCs (Figure 5D).

Notably, some TFs switch function over time, acting as dual regulators or preventive developmental gatekeepers along the genesis of the RPE by accompanying the progression from stemness through RPE cell fate determination to terminal differentiation (*all.summary.xls). Among the identified TFs, some switch regulatory function from repressors to activators of gene expression (Figure 5D) (PBX1, GLI1, GLI2, GLI3, MYCN, LHX2, GATA6, HEY2, TGIF1, and SMAD3), whereas others transition from activators to repressors of gene expression (TWST1, TEAD2, NFIB, TBX3, PLAG1, ZIC1, KLF5, SNAI2, HTF4 i.e., TCF12, and HIC1).

PITX3, STAT3, NFIA, NFIC, MSX1, and MSX2 are primarily functional in the iRPE, where they repress targets that are shared with RPE progenitors and activate those shared with hiPSCs.

CLOCK also activates gene expression in the RPE progenitors compared to hiPSCs first, then turns into a repressor in the transition between RPE progenitors and RPE. The RPE is known to possess a functional circadian clock potentially involved in the regulation of the rhythmic phagocytic activity [25], and impairment of the clock by changes in IL8-mediated melatonin signaling in the elderly may be involved in the pathogenesis of AMD [26].

Select TFBS within the human genome are reported (Table S4) as potential targets for pharmacogenomic therapies.

### 2.4. TFs Regulatory Targets and New Avenues for Pharmacological Intervention on AMD

The Sonic hedgehog (Shh), Wnt/b-catenin, and Notch signaling pathways are involved in the morphogenesis of the RPE, with miRNA expression promoting its differentiation via suppression of the AKT2/(mTOR) signaling pathway [6].

Recent evidence has shown that increased levels of *AKT2*, upregulated in dysfunctional RPE and dry AMD patients [6], impair TFEB/TFE3-dependent lysosomal and mitochondrial function in an iPSC-derived dry AMD model. This occurs through the inhibition of PGC1α, a master regulator of mitochondrial and energy metabolism homeostasis [27], and reduced interaction with neuroprotective SIRT5 [28].

We found that TFEB represses gene expression in differentiated RPE compared to hiPSC and to committed RPE precursors (*all.summary.xls). Its target genes are involved in the regulation of G0 to G1 transition, in the inner mitochondrial membrane organization and membrane potential, as well as increased tumor latency, and *AKT2* was one of its many predicted targets (−53691, TSS). Several footprinted targets were also found to be ontologically enriched for the regulation of autophagy and endosome-to-lysosome transport. Among the TFEB-footprinted genes, we also identified genetic targets involved in the regulation of cell migration in sprouting angiogenesis and in the negative regulation of vasculature development, which have clinical relevance in proliferative diabetic retinopathy and neovascular AMD.

TFE3 is predicted to exert a pioneer function in the transition between committed RPE precursors towards terminally differentiated RPE (*all.summary.xls), activating gene expression involved in transferrin and ferric iron transport, in the intracellular pH reduction, cellular pigmentation, and BLOC complex. TFE3 transcriptional targets identified by footprinting analysis were also found in association with the regulation of G0 to G1 transition and in phagosome acidification and maturation, similar to the TFEB transcriptional repertoire. Hence, gene ontologies were shared between TFEB and TFE3, consistent with previous findings indicating that TFE3 can exert a compensatory function on TFEB loss of function [29].

We also identified several targets for PGC1α (PPARGC1A, peroxisome proliferator-activated receptor gamma coactivator 1-alpha), the loss of which compromises the RPE integrity, mimicking dry AMD [28,30] and we found that PGC1α represses gene expression in iPSCs compared to differentiated RPE while acting, instead, as an activator in committed RPE precursors compared to differentiated RPE (*all.summary.xls). We footprinted PGC1α on genes involved in mitochondrial biogenesis and in the SMAD signaling pathway, in the regulation of phagocytosis, and in the establishment of protein localization to the mitochondrion. Additional gene ontologies include arterial endothelial cell differentiation, adherens junctions assembly, and anoikis, all key RPE functions that are known to be disrupted in the epithelial-mesenchymal (EMT) pathological transformation, proliferative diabetic retinopathy, and AMD.

Furthermore, a widespread decrease in chromatin accessibility is known to affect the ocular tissues of AMD patients [4]: preferential recruitment of specific TFs occurs within differentially accessible regions of macular photoreceptors and the RPE, although their causative role has not been hypothesized yet.

Here, we predict a developmental pioneer function for the AMD-related TFs POU3F3, POU2F1, CUX1, SOX5, PITX2, and CDC5L and identify potential interventional targets.

POU3F3 (BRN1) was footprinted on genes involved in the endothelial cell fate commitment, the insulin metabolism, and cell cycle G2/M phase transition, such as *CDC25C* (−708, TSS), the neurogenic RPE reprogramming factor *NEUROG1* (−154, TSS), the chromatin remodeling factor *BRD4* (−6961, TSS; 1655), and the transcriptional repressor *SIN3A* (−4237, TSS).

POU2F1 (OCT1) was footprinted on genes involved in endothelial cell differentiation, including *ROCK1*, an effector of Rho involved in cell polarity and cytoskeleton remodeling, activated by hyperglycemia and a possible target in diabetic retinopathy, *IKBKB* (−1340, TSS), an activator of the NFKB signaling pathway, and *ADD1* (+827, TSS), involved in cytoskeleton regulation.

CDC5L footprints are associated with genes involved in photoreceptor outer segment degeneration, such as *ABCA4*, *CRB1*, and *RPE65* (−11172, TSS), and in the abnormal retinal ganglion layer morphology, including *ASCL1* (+88, TSS), *CCND1* (+1278, TSS), *POU4F2* (*BRN3B*) (8014, TSS), and *TFAP2A* (2476, TSS).

SOX5 is predicted to regulate gene expression related to cytokine secretion, interferon gamma production, and cell migration involved in sprouting angiogenesis and cellular pigmentation, such as *TLR3* (−704, TSS), *DCT* (−858, TSS), *TYR* (207, TSS), and *BBS4* (849, TSS).

PITX2 predicted targets from footprinting analysis are involved in the production of IL1b, which is known to be induced by inflammatory adipose circuits leading to iron sequestration and overload and, ultimately, RPE degeneration in AMD. Some of the identified targets are *BRD2* (−1482, TSS), *BRD4* (−8980, TSS), *IL10* (5504, TSS), *LDLR* (760; 6233, TSS), *RAB27A* (902, TSS), *TBK1* (−1464, TSS), and *TNFAIP8L2* (−777, TSS). Additional ontologies include the release of cytochrome c from mitochondria, with several targets such as *BAK1* (−558, TSS), *BAX* (−528, TSS), *FIS1* (−7661, TSS), *FZD9* (−421, TSS), *SFN* (2585, TSS), and the reactive oxygen species scavenger *SOD2* (−9866, TSS). Several TFs and chromatin remodelers encoding genes were also targeted by PITX2, such as the enhancer mediator protein *MED1* (−409, TSS), the histone deacetylases *HDAC4* (−7317, TSS), *HDAC7* (−6175, TSS), *HDAC11* (5848, TSS), the histone acetyltransferase *HAT1* (−787, TSS), the chromatin scaffold protein *WDR5* (−5626, TSS), and the methyltransferase and chromatin repressor *SETDB1* (−537, TSS) (Table S4).

### 2.5. Predicted Pioneer Factors and Their Regulatory Targets Are Associated with Latent Developmental Pathways or Alternate Neuro-Mesodermal Cell Fates and the Epithelial-Mesenchymal Transition

Several predicted pioneer factors (Table S3) and related regulatory targets (Table S4) were found in association with latent developmental pathways or alternate neuro-mesodermal cell fates, as they are known to be involved in the cell fate specification of the hematopoietic, osteogenic, chondrogenic, adipogenic, and myogenic lineages.

First, neuroepithelial TFs and determinants of mesodermal derivatives were among the predicted repressors of gene expression in the transition between iPSCs and RPE. Master regulators include KLF9, SRF, HES1, and SMARCA5 (SMC5A), with EPAS1 (HIF2A) and SUH (RBPJ) identified in the transition between RPE progenitors and differentiated RPE. Some repressors, instead, were preferentially functional in hiPSCs compared to differentiated RPE, such as ETV3, FOXJ3, GLI3, HTF4 (TCF12), NFAT5, and STAT1, and others were active in the transition between hiPSCs and RPE progenitors, such as MSX2, FOXJ3, FOXC1, and FOXH1 (Table S3).

Second, some TFs are involved in the regulation of hemopoiesis and methylation of CpG islands, potentially related to the reset of the epigenetic memory or somatic reprogramming. While the KAISO (ZBTB33) repressor was preferentially functional in hiPSCs compared to RPE progenitors, DNA methylation factors such as MECP2, MBD2, and WT1 were identified as repressors of gene expression in the RPE compared to iPSCs. SUH (RBPJ), VEZF1, ZBT7A (ZBTB7A), and ZFX were also identified among the iRPE developmental repressors; they are known to be involved in the regulation of DNA methylation through the expression/recruitment of DNA methyltransferases/demethylases, and they may control the timing of cell lineage differentiation.

Additionally, we identified several drivers of pathological epithelial-mesenchymal transition (EMT) and related binding targets.

The EMT is a process by which the retinal pigment epithelium loses apicobasal polarity, morphology, adhesiveness, and cell-to-cell contact while transdifferentiating into myofibroblasts, acquiring invasiveness and the ability to proliferate. It is a hallmark of proliferative vitreoretinopathy, diabetic retinopathy, and AMD [31]. Several studies have shown that RPE cells can undergo epithelial-myofibroblast transition, migratory changes, and pigmentary abnormalities in AMD [32], with subretinal fibrosis as an end-stage manifestation of wet or neovascular AMD (nvAMD) [31]. Extracellular matrix deposition occurs as a wound-healing mechanism in response to choroidal neovascularization in nvAMD, accounting for 10–15% of all cases. Dysregulated metabolism of the RPE and persistent inflammation have been implicated in the pathogenesis of AMD, involving the production of cytokines such as TNFα, TGFβ2, IL6, and IL1β, which, in turn, exacerbate tissue damage, mitochondrial uncoupling through electron leakage, oxidative stress, fibrosis, and necrosis [33]. TNFα and TGFβ2 increase lactate production, glycolysis, and O_2_ consumption, leading to the activation of the PI3K/AKT and the MEK/ERK pathways.

An additional context where the EMT plays a pathological role is tumor metastasis and chemoresistance [34,35]. SNAIL, TWIST1, ZEB1, and bHLHs promote the expression of proteolytic enzymes, hence extracellular matrix breakdown and extravasation, propagation, cellular resistance to apoptosis and senescence, modulating cellular response to drugs. Notably, SNAILs, ZEB, and bHLH family members have also been shown to drive the EMT transformation of the RPE under inflammatory conditions [36]. Unfolded protein response and TGFB-induced EMT signaling pathways activate the JNK/p38-MAPK pathway [37,38], leading to the upregulation of *SNAIL* and *ZEB* family members. SMAD effectors are also responsible for the induction of known EMT master regulators under chronic inflammation of the RPE as a result of increased production of TGFB [39,40], whereas inhibition of TGFB and FGF/MAPK pathways has been shown to improve iPSC-derived RPE differentiation efficiency [41]. A previous study profiling human stem cell–derived RPE induced to EMT by enzymatic dissociation and TGFβ/TNFα treatment had shown transcriptional similarities between de-differentiating RPE and a metastatic model of cancer, with *SNAI1* and *SNAI2* mRNAs upregulated shortly after mechanical dissociation, followed by *ZEB1* and *TWIST1* (*TWST1*) [42]. In that study, Notch signaling effectors were also upregulated, such as *HES1*, *HEY1*, and *JAG1*, as well as *TGFB1*, *NFKB1*, *HMGA2*, and *CTNNB1*. In fact, transcriptional comparison with a gland tumor model had revealed recurring genes involved in the EMT process, cell migration, focal adhesion, and integrin complex formation [42], as well as the deregulation of axon guidance molecules, fibrosis, the IL8 pathway, and genetic targets of cancer-associated TFs such as NFKB1, STAT3, RELA, JUN, and HIF1A (Appendix A).

Here, we predict multiple pioneer factors in the transition from hiPSCs to RPE involved in the EMT signaling pathways, wound healing, and myofibroblast differentiation (Figure 5D, Table S3), as well as axonogenesis and cellular response to oxygen-containing compounds (Table S3). By taking into account all TFs known to be involved in the EMT process (i.e., statistically de-regulated mRNAs or inferred from promoter analysis of statistically de-regulated target genes), we found many noticeable examples of EMT-induced TFs [42] for which we predicted a pioneer function in the RPE developmental context (Appendix A).

In fact, several TFs do bind to known EMT targets, as revealed by footprinting analysis of extended regulatory regions.

First, among the identified developmental iRPE repressors, several are known to regulate tumorigenesis, such as COT1 (NR2F1), KLF12, MXI1, NR2F6, PLAL1 (PLAGL1), PBX1, PBX3, ZNF263, ZBTB6, and ZBT18, with TAF1 involved in the regulation of telomere stability. Additionally, BHLHE41, CDC5L, FOXC1, HIC1, KLF3, SOX13, TBX3, and TEAD2 have been described in metastatic EMT type III contexts.

Second, the iRPE repressors identified in our screening are involved in axonogenesis regulation, such as KLF9, KLF13, and LHX2 (downregulated at increasing dosages in the TGFβ/TNFα EMT model). LHX2 was footprinted on extended regulatory regions of semaphorin genes (negative regulators of axonogenesis), on genes involved in the abnormal retinal ganglion cell morphology and degeneration, and hypopigmentation (Table S4): these ontologies are known to be involved in the EMT pathogenic transformation of the RPE [42].

Third, we identified several oxidative stress-related TFs among the iRPE repressors, such as ATF2 and ATF4, ARNT2, CREB3 and CREB3L2, EGR1, EPAS1 (HIF1A), ESRRA (ERR1), HMGA1, and KLF9.

Finally, we also identified the corresponding TF-specific binding sites within proximal promoter regions, likely direct regulatory targets of gene expression within the human genome (Table S4), such as the downstream targets of the canonical TGFβ/Smad, Hippo/YAP, and MAPK pathways and related effectors (Smad2/3 and p38 MAPK). For instance, the *SMAD3* gene, a key mediator of the TGFB-induced EMT process, was footprinted by several predicted pioneering factors (FOS, FOSL2, TEAD2, CEBPB, MEIS2, TBX3, GATA6, JUN, PBX1, GLI3, TWIST1, EGR1, PBX3, ASCL1, NFIB, PBX1, STAT3, and NFKB1).

ZEB1 and PBX1, affected in EMT models [42], activate gene expression in RPE progenitors compared to RPE. We found that the predicted EMT drivers ZEB1, SNAI1, SNAI2, TWIST1, SMAD3, and HIC1 act as preferential repressors in hiPSCs compared to RPE (Figure 5D; *all.summary.xls) under physiological conditions.

## 3. Discussion

### 3.1. Dual Regulators and Preventive Developmental Gatekeepers Suppress Latent, Alternate Neuro-Mesodermal Cell Fates in the Consolidation of the Human RPE Cell Fate Identity

Here, we screened open chromatin along three stages of human-induced RPE genesis from pluripotency, through RPE-committed precursors, to functionally tested, terminally differentiated RPE. We set out to identify all predicted pioneer and regulatory factors in the human RPE epigenome that modulate chromatin accessibility by either priming it for gene expression or inducing its compaction, leading to gene repression. Hence, we identified biological functions regulated by such TFs.

Empirical detection of all TF binding signatures, computation of chromatin accessibility, TF footprinting analysis, and stage-matched variations in gene expression in paired developmental stages led to the prediction of TF activators and repressors along the human RPE developmental trajectory and related ontologies, leveraging a pipeline that was previously shown to correlate with ChIP-Seq validated TFBS [16].

First, neuro-ectodermal progenitors from all three donors were consistently positive for stemness and neuroepithelial markers, although we detected differences in the efficiency of iRPE generation among batches, leading to different neuroepithelial derivatives. It is possible that higher efficiency in the inhibition of the FGF pathway, activation of the TGF pathway or the canonical WNT pathway, as well as intrinsic biases towards an RPE cell fate, may drive preferential commitment towards it.

Sustained expression of some predicted pioneer factors (OTX2, LHX2, MEIS2, PAX6, NFIB, and SMAD3, to name a few) seemed propitious for RPE cell fate specification, consistent with previous evidence in murine models indicating that small endogenous fluctuations of pioneer factors can bias cell fate decisions by concentration-dependent priming of differentiation-associated enhancers, impacting the efficiency of mesendodermal and neuroectodermal commitment [43]. Indeed, we identified many neuroepithelial TFs with predicted pioneer function, as well as determinants of mesodermal derivatives involved in hemopoietic, osteogenic, adipogenic, cardiac, and skeletal muscle cell fate specification, which act as repressors of gene expression in the transition between hiPSCs to RPE and are preferentially functional in human stem cells. It is plausible that these TFs may deflect cell fate specification from alternate developmental pathways by channeling stem cells towards an RPE cell fate.

Second, some of the repressors are involved in the regulation of hemopoiesis and methylation of CpG islands in the transition between iPSCs and RPE, potentially related to the reset of the epigenetic memory or somatic reprogramming. DNA methylation factors such as KAISO (ZBTB33), MECP2, MBD2, and WT1 were also identified as repressors of gene expression, as well as SUH (RBPJ), VEZF1, ZBT7A (ZBTB7A), and ZFX, providing an additional tool to suppress alternate cell fates while consolidating the RPE epigenetic identity. These findings suggest that default suppression of latent, alternate neuro-mesodermal cell fates may occur during RPE genesis and is maintained, under physiological contexts, once cell fate determination of bipotent neuroepithelial progenitors has taken place. Hence, suppression of alternate cell fates may occur through the expression/recruitment of DNA methyltransferases/demethylases or direct CpG methylation of relevant neurogenic and mesenchymal TF-associated genes.

Third, we found that some TFs repress gene expression in hiPSCs compared to the RPE first, then activate gene sets in RPE progenitors compared to the RPE, acting as dual regulators or preventive developmental gatekeepers under physiological conditions. It is plausible that dual TFs may prevent precocious differentiation of neuroectodermal progenitors, allowing for proper timing of cell fate determination.

Some of the neuroepithelial and mesenchymal determinants that emerge from our screening, such as predicted pioneer factors MYOD1, MYOG, MEF2B, MEIS2, ATOH1, or FOXD1, display negligible mRNA expression in the RPE context. While their biological significance remains to be assessed, it is plausible that either homologs with similar motif recognition or minimum amounts of translated proteins with low turnover rates may suffice to repress alternate neuro-mesodermal cell fates so as to ensure the consolidation of the RPE cell fate.

Targeted biochemical methods allow assessment and quantification of protein-DNA and protein-nucleosome binding affinity to unequivocally identify pioneer factors. These range from comparative, semi-quantitative in vitro nucleosome reconstitution and binding assays to electrophoretic mobility shift assays (EMSA), supershift assays, and SILAC nucleosome affinity purification methods (SNAP). Recent implementations can simultaneously determine apparent dissociation constants of multiple nuclear proteins from DNA and from nucleosome ligands so as to accurately and quantitatively profile the proteome binding landscape with nucleotide resolution and in post-translationally defined conditions [44] for precise substrate recognition within multimeric complexes. Fluorescence resonance energy transfer (FRET), affinity purification of native nucleosomes combined with UV-mediated DNA–protein cross-linking, isotopic and isobaric labeling, and SILAC-based nucleosome affinity purification for quantitative mass spectrometry [44] are now enabling an unbiased, systemic, and quantitative interrogation of the proteome-wide regulatory landscape across genomes.

Our results will complement the aforementioned biochemical and proteomic methods for the empirical validation of pioneer factors and advance the understanding of the molecular and epigenetic mechanisms behind the human RPE cell fate specification and homeostatic tissue maintenance so as to inform new therapeutic directions for the RPE regeneration and repair.

### 3.2. Pharmacogenomics to Restore the RPE and Pleiotropic Cis-Regulatory Elements in Complex Diseases

The epithelial-mesenchymal transition (EMT) is common to proliferative vitreoretinopathy, diabetic retinopathy, and AMD, and it has also been described in other developmental and pathological contexts, such as wound healing in tissue regeneration and metastatic transformation in cancer.

As we screened for developmentally active pioneer factors along the human RPE genesis, we discovered that many TFs had been previously identified in experimental paradigms of RPE damage [42]. Some have been implicated in the regulation of axonogenesis (FOSB, GLI3, GLI2, HES1, ATF3, ATF4, and KLF9), in the cellular response to oxidative stress (TWIST1, BACH1, FOXP1, GLI1, MSX1, EGR1, HIF1A, and HMGA1), and in tumorigenic, or type III EMT (NFKB1, STAT3, RELA, JUN, GLI3, GLI2, and HIF1A, to name a few). TF rewiring due to a combined effect of haplotype predisposition, environmental factors, and reactivation of developmental pathways may contribute to the pathogenesis of the RPE.

First, we found that ZEB1, SNAI1, SNAI2, TWST1, SMAD3, and HIC1, known to be affected upon EMT in both the RPE and forms of cancer, are preferential repressors in hiPSCs compared to RPE, likely acting as preventive developmental gatekeepers under physiological conditions. While these predicted EMT drivers have not been tested in the present work, we hypothesize that these developmental TF regulators may play a role in the pathogenesis of the RPE: the EMT may reactivate cell cycle genes and early developmental pathways or alternate neuroectodermal cell fates that are normally suppressed in the RPE specification context. Pleiotropic targets that are evoked during inflammatory and degenerative conditions of the RPE, such as binding sites for the predicted EMT drivers SNAIL1/2, TWIST1, ZEB, and bHLH family members, may provide potential avenues to address subretinal fibrosis in nvAMD, proliferative diabetic retinopathy, and, potentially, chemoresistance in cancer via pharmacogenomic, targeted therapies. Furthermore, the expression level for some TFs with predicted pioneer function is known to vary rapidly as soon as the EMT process is initiated, whereas others are only affected at high dosages of TGFβ/TNFα treatment, suggesting possible strategies to differentiate EMT treatment: Early intervention or preventive strategies against EMT could target the genetic repertoire of “early response TF genes” first. Subsequent intervention would focus, instead, on regulatory regions bound by (or responsible for promoter expression of) “late response TFs (genes)” that are induced at later stages of disease progression (Appendix A).

Second, we provide insights into possible causative mechanisms of AMD, where a widespread decrease in chromatin accessibility is known to occur [4]: while preferential TF recruitment within differentially accessible regions of the RPE had been reported in AMD patients, the role of such TFs has not been further investigated.

Here, not only do we predict a developmental pioneer function for POU3F3, POU2F1, CUX1, SOX5, PITX2, and CDC5L, but we also identify potential interventional targets. Besides the histone deacetylases HDAC4, HDAC7, and HDAC11, we pinpoint previously uncharacterized epigenetic factors that could be involved in the pathogenesis of AMD, such as the transcriptional repressor SIN3A, the enhancer mediator protein MED1, the histone acetyltransferases HAT1, BRD2, and BRD4, the chromatin scaffold protein WDR5, and the methyltransferase and chromatin repressor SETDB1.

Additional targets with pharmacological relevance for AMD are regulators of cholesterol biosynthesis, lipid homeostasis, and metabolism, since drugs that promote lipidic clearance and APOE-mediated cholesterol efflux have shown promising results in vitro and in vivo [9]. It is worth noticing that cholesterol and apolipoproteins are common etiological factors in progressive neurodegenerative disorders and chronic diseases such as AMD, Alzheimer’s disease, and atherosclerosis [9,45]. Genome-wide association studies have identified SNPs and pleiotropic genes contributing to the comorbidity and shared etiology of AMD and Alzheimer’s disease, such as APOC1 and APOE, along with potential upstream regulators ZNF131, ADNP2, and HINFP [46], a factor for which we predicted a pioneer function in the transition between RPE progenitors and terminally differentiated RPE. We found that the APOE human promoter and its extended regulatory regions are footprinted by GATA6, BACH1, and predicted pioneer factors ASCL1, PBX1, ZIC1, and NFIB. Among the identified iRPE developmental repressors, we also identified the adipogenic factors SREBF1 and SREBF2 (SRBP2), as well as COT2 (NR2F2) and ERR1 (ESRRA).

Alternate routes for intervention and restoration of the RPE entail cytosolic sequestration of EMT signals by tight and adherens junctions, supplementation with miRNAs-204/211, nicotinamide, and antioxidant flavonoids, regulation of the PI3K/AKT/GSK-3β axis for cytoprotection, as well as clearance from reactive oxygen species (ROS) for reduced lipid peroxidation. NRF2, for example, is a known EMT preventive factor, and it is involved in cytoprotection from ROS [47]. Notably, NRF2 and peroxiredoxin PRDX6 exert protective effects from moderate oxidative damage in human lens epithelial cells [48] and in the human RPE, where they preserve the differentiated phenotype, preventing EMT transformation in a dry AMD model [30,49]. We identified several predicted pioneer factors in the transition between hiPSCs and RPE that are involved in cellular responses to oxygen-containing compounds, such as the iRPE repressors ATF2 and ATF4, ARNT2, CREB3 and CREB3L2, EGR1, EPAS1 (HIF1A), ESRRA (ERR1), HMGA1, and KLF9, which is known to exacerbate hypoxia-induced neo-angiogenic responses relevant to nvAMD and proliferative diabetic retinopathy. Hence, identifying sensors of oxygen and environmental fluctuations and understanding hormetic responses to oxidative stress that are triggered by sub-pathogenic doses of ROS [48] may help elucidate endogenous adaptive mechanisms of the RPE and homeostatic resilience to stress.

To summarize, this work identifies potential interventional targets, setting the ground for therapeutic strategies that aim to (i) modulate the complement system and the inflammatory response via pharmacogenomic inhibition of the TGFβ, SMAD2/3, Hippo/YAP, MAPK/p38, and WNT signaling pathways; (ii) promote mitochondrial integrity, increase lipoprotein expression, and facilitate cholesterol and ceramide clearance to prevent lipoprotein phase separation and drusen nucleation; (iii) prevent iron accumulation and ferroptosis; (iv) identify and leverage hormetic, protective responses to oxidative stress.

By leveraging our compendium of TF-specific binding sites identified in a developing hiPSCs-derived RPE model, it will be possible to assess which cis and trans regulatory sites in the human genome are necessary and sufficient to consolidate and preserve the epithelial morphology under physiological conditions, to tentatively repopulate the native RPE, or to revert the mesenchymal phenotype to an epithelial state prior to the nuclear translocation of EMT drivers. 

Recent advances in scalable single-cell quantitative expression reporters via RNA barcode stabilization and circularization are now enabling scalable screening of hundreds of accessible chromatin domains in multicellular in vitro models of early mammalian development. Epimutations can be engineered via conjugation of dCas9 with chromatin effectors to achieve reversible gene activation/silencing and higher-order chromatin perturbations. CRISPR-Cpf1, an enhanced CRISPR-iCas12a system, prime editing with circular RNA nickase, and cytosine- and adenine-based editors are leading to multiplexed genome editing, mutagenesis, genetic knock-in/out phenotyping, and disease modeling without introducing DNA breaks.

Simultaneous perturbation of multiple regulatory elements may enable selective and combined control of signaling transduction effectors involved in the EMT transition of the RPE while leaving the expression levels for the TF of interest unaffected, hence limiting off-target effects or systemic disruption of signaling cascades.

We anticipate that this comprehensive repository of predicted pioneer factors and transcription factor binding sites within the human RPE epigenome will serve as a reference for functional screenings of pleiotropic regulatory elements that are dysregulated in several pathological conditions. As such, it may help devise pharmacogenomic therapies intended to rescue vision loss in RPE degenerative conditions and, potentially, chemoresistance in type III EMT contexts, paving the way for new strategies in precision medicine.

## 4. Materials and Methods

### 4.1. Human iPSCs Derivation, Phenotyping, and Reprogramming to Retinal Pigment Epithelium

Following density gradient centrifugation of whole blood and retention of PBMCs, immunomagnetic purification of HSCs was performed according to the manufacturer’s protocol (Miltenyi Biotec, Bergisch Gladbach, Germany, CD34 MicroBead Kit UltraPure, human), and cell eluates were assessed by flow cytometry with CD45 (BD Pharmingen™, Milpitas, CA, USA, 560274), CD34 (Miltenyi Biotec, Bergisch Gladbach, Germany, 130-120-520), and CD90 (Miltenyi Biotec, Bergisch Gladbach, Germany, 130-117-796).

Peripheral blood mononuclear cells (PBMC) were extracted from three healthy donors, purified for CD34+ hematopoietic stem cells by immunomagnetic retention, expanded for 72 h in the presence of IL-3, IL-6, SCF, and TPO, and reprogrammed by nucleofection with non-integrating episomal plasmids, and iPSC derivation was performed as described previously [50,51,52,53,54]. Residual expression of transgenes was assessed across passages and monitored through the loss of oriP, present in every vectorial backbone, prior to RPE induction.

All derivative iPSC clones were expanded and phenotypically profiled by q-RT-PCR, including 4 primary subclones and 9 time points at increasing passage for the cell line derived from donor 1, 6 time points for the cell line derived from donor 2, and 6 time points from different cell lines derived from donor 3. The expression of the endogenous, canonical stem cell markers was validated, and further neuro-ectodermal, mesodermal, and endodermal markers were tested (mean 2^(−∆∆Ct)^, i.e., 2-DDCt, GAPDH as housekeeping gene) compared to PBMC prior to reprogramming. iPSC batches were subject to oncogene exome sequencing.

RPE induction was carried out as previously described [10,55], (ongoing NIH clinical trial ID: NCT04339764). All cells were cultured in a cell culture incubator at 37 °C and 5% CO_2_. Depending on the stage of cell development, different cell media were used [56]. Briefly, iPSCs were seeded in neuro-ectoderm induction Medium (NEIM)—DMEM/F-12, KOSR (KnockOut™ SR XenoFree, Life Technologies, Bleiswijk, The Netherlands), supplemented with 1% (mass/volume) N-2, B-27, LDN-193,189 10 μ mol/LM, SB 431452 10 nmol/L, CKI-7 dihydrochloride 0.5 μ mol/L, and IGF-1 1 ng/mL. Neuro-ectodermal progenitors were seeded on vitronectin; after 2 days, cells were transferred to RPEIM (DMEM/F12, KOSR, supplemented with N2, B27, LDN-193189 100 uM, SB431452 100 nM, CKI-7 hydrochloride 5 uM, and IGF-1 10 ng/mL, PD0325901 1 uM) for 10 days and then to RPECM (DMEM/F12, KOSR, supplemented with N2, B27, Nicotinamide 10 mM, and Activin A 100 ng/mL) for another 10 days. On day 22, cells were switched to RPEGM (MEM; 1% N2 supplement; 1% Glutamine; 1% non-essential amino acids; 125 mg Taurine/500 mL; 10 ug Hydrocortisone/500 mL; 0.0065 ug Triiodo-thyronin/500 mL; 5% FBS), dissociated in Versene at day 27, and reseeded in RPEGM. On day 42, cells were dissociated in TrypLE and re-seeded in RPEMM (MEM; 1% N2 supplement; 1% Glutamine; 1% non-essential amino acids; 125 mg Taurine)/500 mL; 10 ug Hydrocortisone/500 mL; 0.0065 ug Triiodo-thyronin/500 mL; 5% FBS, Sigma-Aldrich, Saint Louis, MO, USA origin, sterile-filtered [F2442]; 50 uM PGE2) onto 6-well 0.4 μm transwell plates.

Neuroepithelial derivatives progress to an RPE-primed state following hiPSCs mesenchymal-epithelial transition [57,58]. The emergence of stratified differentiative niches and pigmented halos was observed during the first month of iRPE genesis. Concentric differentiative niches of RPE progenitors surrounded by fibroblast-looking cells were also observed, as previously described [59,60], with evenly distributed, cuboidal cells by the third month of iRPE induction.

Differentiated RPE can be retained upon subsequent but limited passages before de-differentiation or phenotypic EMT switch occurs, as previously shown [39,42].

### 4.2. RPE Phenotyping

Immunostaining of iPSCs and derivative RPE was performed as follows. Briefly, cells were fixed in 4% PFA at room temperature for 15 min, permeabilized for 2 h in 0.2% Triton X-100 at room temperature, and incubated overnight at 4 °C or for 4 h at room temperature, depending on the manufacturer’s recommendations, washed three times, then incubated in secondary antibody for two hours at room temperature, with the following antibodies: NANOG (BD Pharmingen^TM^, Erembodegem, Belgium, 560791), TRA-1-60 (BD, Pharmingen^TM^, Milpitas, CA, USA, 560122), SSEA5 (Miltenyi Biotec, Bergisch Gladbach, Germany, 130-106-722), OTX2 (AbCAM, Amsterdam, The Netherlands, ab9566), OCT4 (AbCAM, Amsterdam, The Netherlands, Ab79857), VE-cadherin (AbCAM, Amsterdam, The Netherlands, ab7047), Nestin (Santa Cruz Biotechnology, St. Louis, MO, USA, sc23927), TuJ1 (AbCAM, Amsterdam, The Netherlands, ab7751), PAX6 (BioLegend Inc.,San Diego, CA, USA, 901302), DCT (ThermoFisher Scientific, Waltham, MA, USA, PA5-105275), RPE65 (Invitrogen, ThermoFisher Scientific, Waltham, MA, USA, PA5-110315), ZO1 (ThermoFisher Scientific, Rockford, IL, USA, 33-9100), MITF (ThermoFisher Scientific, Fremont, CA, USA, MS-771-P1), BEST1 (Novus Biologicals, Centennial, CO, USA, NB300-164SS), ezrin (Biosite, Irvine, CA, USA, GTX-111709), and AF-647 conjugated acquaporin (AbCAM, Amsterdam, The Netherlands, ab225225). Secondary antibodies are AF488 donkey anti-rabbit (Invitrogen, ThermoFisher Scientific, Rockford, IL, USA, A21206), AF488 goat anti-mouse (Invitrogen, ThermoFisher Scientific, Bengaluru, India, A28175), AF594 goat anti-rabbit (Invitrogen, ThermoFisher Scientific, Bleiswijk, The Netherlands, A11012), and Cy-3 goat anti-mouse (Invitrogen, ThermoFisher Scientific, Rockford, IL, USA, A10521).

Characterization was also compared to [61,62,63,64,65]. Sandwich VEGF and PEDF ELISA was performed according to the manufacturer’s protocols (DuoSet ELISA human VEGF and PEDF, R&D Systems, Biotechne, Minneapolis, MN, USA; Victor Multimode plate reader). PEDF is primarily secreted towards the apical compartment before full differentiation of RPE is achieved, as previously described [66].

Transepithelial resistance measurements were performed as previously described (Millicell^®^ ERS-2 Voltohm-meter, EMD Millipore Corporation, Billerica, MA, USA) [67]. Images were acquired on a Zeiss LSM 880 microscope (Zeiss, Jena, Germany).

### 4.3. Next-Generation Sequencing

ATAC-Seq and RNA-Seq libraries were generated as described previously [4,19]. All libraries were subject to quality controls and run on an Illumina NovaSeq 6000 platform. All samples were preliminarily quality controlled by Qubit™ dsDNA High Sensitivity and Qubit™ RNA High Sensitivity assays (Invitrogen, Life Technologies, Carlsbad, CA, USA) prior to library preparation.

ATAC-Seq libraries were generated with the Illumina Tagment DNA Enzyme and Buffer kit (Illumina, San Diego, CA, USA, 15027866), quantified by Qubit dsDNA Broad Range (Invitrogen, Life Technologies, Carlsbad, CA, USA), run on an Agilent TapeStation High Sensitivity D1000 ScreenTape or on an Agilent Bioanalyzer 2100 with DNA ChIP 1000 kit and DNA High Sensitivity kit (Agilent Technologies, Waldbronn, Germany) for smear analysis, and purified by solid phase reversible immobilization prior to pooling according to the Illumina’s barcoded indexes recommendations (Document # 1000000002694 v10).

Specifically, 150 bp paired-end libraries were lane run (800 Gb) on the Illumina NovaSeq6000 platform (PE150, Phred score Q30 [99.9% accuracy] ≥ 82%), resulting in sequencing depths from 50 M to 500 M reads per sample. Next-generation sequencing was run by Novogene, Cambridge, UK.

Directional RNA library preparation (rRNA removal, lincRNA-Seq) was performed upon QC assessment by Qubit RNA HS kit (Invitrogen, Life Technologies, Carlsbad USA), RIN checked (≥8) according to Novogene pipelines, and 150 bp paired end libraries were run on the Illumina NovaSeq6000 platform with a minimum sequencing depth of 36 Gb of raw data per sample (120 M reads circa) (PE150, Phred score Q30 [99.9% accuracy] ≥ 85%). Burrows–Wheeler Aligner (BWA) v0.7.17 was used to map the paired-end clean reads to the hg38 reference genome (http://hgdownload.cse.ucsc.edu/goldenPath/hg38/bigZips/analysisSet/hg38.analysisSet.2bit (accessed on 31 March 2022))

The original mapping results were obtained in BAM format. SAMtools v1.8 [68] was used for sorting the BAM files, and Picard v2.18.9 [69] was utilized to mark duplicate reads.

For RNA-Seq libraries, ribosomal RNA was removed by the rRNA removal kit and purified by ethanol precipitation. Subsequently, sequencing libraries were generated using the rRNA-depleted RNA and performing procedures as follows. Briefly, after fragmentation, the first strand of cDNA was synthesized using random hexamer primers.

Then, the second strand of cDNA was synthesized, and dUTPs were replaced with dTTPs in the reaction buffer. The directional library was ready after end repair, A-tailing, adapter ligation, size selection, enzyme digestion, amplification, and purification. Libraries were screened by Qubit, real-time PCR for quantification, and bioanalyzer for size distribution detection (see above).

Cuffdiff [70] was used to calculate FPKMs of coding transcripts in each sample for differential expression analysis. Transcripts with P-adjust < 0.05 were described as differentially expressed between any paired comparison and profiled as differentially expressed transcripts, cross-referenced against the GO Ontology and Kyoto Encyclopedia of Genes and Genomes (KEGG) [71].

DNA samples intended for whole-exome sequencing (WES) were quantified by Qubit dsDNA Broad Range, the related libraries prepared with the DNA library preparation Agilent SureSelect Human All Exome V6 (58 M) kit, and run as paired-end 150 bp on the Illumina NovaSeq6000 platform with a minimum sequencing depth of 12 Gb raw data per sample (PE150, Phred score Q30 [99.9% accuracy] ≥ 85%) at 100x coverage. SNP and InDel calling, annotation, and statistics were performed, as well as somatic variant (SNP, InDel, and CNV) calling, annotation, and statistics for paired samples (PBMCs vs. reprogrammed hiPSCs). Picard tools [69] and Samtools [68] were used to sort, mark duplicated reads, and reorder the BAM alignment results of each sample. Then, the tool HaplotypeCaller in GATK software v4.0 [72,73] was used to perform variant discovery, including SNPs and INDELs. Raw VCF files were filtered with the GATK standard filter method and other parameters (cluster: 3; WindowSize: 35; QD < 2.0 or FS > 30.0). Finally, ANNOVAR v.2015Dec14 [74] was used to functionally annotate genetic variants detected from diverse genomes with user-specified versions of genome builds. The data quality summary is in Table S1. All paired-end libraries were subject to index demultiplexing, adapter removal, data quality controls (Q20, Q30, and GC content calculated), and data filtering, aligned onto the reference human genome hg38 (GRCh38.p13) by HISAT 2 [75], and annotated (Gencode v. 40).

### 4.4. De Novo and Known Motifs Enrichment

Known and novel TF motifs enrichment was first performed on broad ATAC-Seq regions (MACS v2.2.7.1) [76] (callpeak --format=BAMPE --keep-dup=all --gsize=hs –qvalue=0.01 --broad --broad-cutoff=0.05 --cutoff-analysis), then on DNAse peaks called by Homer v4.11 (findPeaks.pl -style dnase -tbp 1) [77] reflecting accessible (open) chromatin regions with interspersed nucleosome gaps. Peaks from experimental replicates were merged (-d given) [77] by physical overlap (26,547 peaks in iRPE, 7738 in functionally tested RPE progenitors, 4740 in RPE progenitors that failed to terminally differentiate and 7416 in hiPSCs), and scanned for motif enrichment, confirming TFs previously identified in broad regions hitting a larger fraction of the genome. Motif enrichment was carried out by binomial scoring of novel oligonucleotides (findmotifGenome.pl; Homer v4.11) [77], TF logos are displayed, together with Pearson’s correlation reflecting similarity to known deposited position weight matrices (PWMs), as previously described [19]. Motif enrichment was carried out on repeats masked hg38 genome patch 13 (GRCh38.p13), at an empirically determined size, 8–15 mer in length, with the top 10 instances optimized and compared with known motifs in the JASPAR 2022 nonredundant core motifs collection [78] for de novo checking.

### 4.5. Gene Ontology Enrichment on Open Chromatin

Nucleosome-free regions were called by Homer v4.11 (findPeaks.pl -style dnase -tbp 1 -nfr) [77], reflecting 150 bp-sized accessible (open) chromatin regions flanked by nucleosome gaps (Figure S6B,E,H). Genome-wide reads histograms normalized by library size were generated with Homer v4.11 [77] (annotatePeaks.pl peaks GRCh38.p13 -size 3000 -hist 50 -ghist), subject to K-means clustering (10 Ks, 100 iterations set), and the identified clusters were analyzed for gene ontology (GO) enrichment with GREAT (Table S2) as described previously [79,80]. Assignment of chromatin regions to putatively regulated genes is not limited to the nearest promoter; rather, it is based on physical overlap with extended promoter regulatory domains. Briefly, all promoters that open chromatin peaks were assigned to, and that populate GOs categories, were defined by extending TSS regions 5000 bp upstream and 1000 bp downstream for up to 1,000,000 bp max extension. Binomial rank was computed for the enriched GOs, and significance was corrected by multiple Hp testing (Bonferroni-Hochberg).

#### Limitations of the Study

Candidate regulatory loci are not functionally tested in the present work. Recently developed quantitative expression reporter methods may ascertain the functionality of candidate regulatory loci by scalably and precisely measuring the activity of developmental cis-regulatory elements (CREs) in multicellular systems [81].

Improvements of cytosine- and adenine-based editors [82] allow multiplexed genome editing, mutagenesis, genetic knock-in, knock-out phenotyping, and disease modeling without introducing DNA breaks. Modifications of the CRISPR interference (CRISPRi) system with multiAsCas12a-KRAB fusion may enable reversible gene activation/silencing and higher-order chromatin perturbations [83].

### 4.6. Detection of Differential Transcription Factor Activity in Human iPSC-Derived RPE Cell Fate Determination and RPE Differentiation

Peaks were called with the following flags (MACS v2.2.7.1) [76]: callpeak --format=BAMPE --keep-dup=all --gsize=hs --qvalue=0.01 --broad --broad-cutoff=0.05 --cutoff-analysis.

Differential activity of Transcription factors (TFs) was computed by paired comparison of cell types along the RPE differentiation trajectory: (1) hematopoietically derived iPSCs versus Intermediate stage (presumptive RPE progenitors, i.e., RPE committed precursors) (2) RPE committed precursors versus terminally differentiated RPE (3) iPSCs versus terminally differentiated RPE.

The framework described in [19,84,85] and implemented in DiffTF [16] relies on the following assumptions: (i) Increased RNA-Seq level for the TF activator should result in its increased abundance, increased usage of its binding sites (footprints), consequently increased chromatin accessibility at the related regulatory elements, and upregulation of its target genes carrying such elements. (ii) Increased RNA-Seq level for the TF repressor should result, instead, in decreased chromatin accessibility at the related position weight matrices (PWMs) and consequent downregulation by RNA-Seq of its target genes.

TFBS predictions are inherently noisy and known to lead to false-positive detections. Nevertheless, DiffTF aggregates signals across thousands of TFBS for each TF. Its technical robustness had been previously assessed by comparing results with ChIP-Seq validated TFBS, which showed strong correlation (Pearson’s R = 0.84) [16]. Motif models of reference are extracted from the HOmo sapiens COmprehensive MOdel COllection (HOCOMOCO) [86], and they are derived from ChIP-Seq and HT-SELEX data by DNA motif discovery, manually curated and benchmarked.

The first analytical step entails ATAC-Seq identification of peaks and identification of PWMs, that is, in silico prediction of putative Transcription Factor binding sites (TFBS).

Differential TF activity is then computed (footprinting) with a *p*-value for each TF, where the activity is displayed as log2 fold change in chromatin accessibility for each TFBS. The significance is assessed using an analytical procedure, which provides a *p*-value based on the t-statistic and estimated variance [16].

In classification mode, putative activators and repressors are identified as follows: DiffTF leverages RNA-Seq labels to estimate the abundance of each TF and that of TF target genes displaying unused TFBSs (PWMs) and used ones (footprints). TF activity represents effective TF binding (footprinting).

First, for every TF, TF-specific expression levels are correlated with the chromatin accessibility of TF-specific target sites compared to non-targeted peaks. If the correlation between the expression level of any given TF and the chromatin accessibility variation of its own target peaks (foreground) is more positive than the correlation computed in the background distribution (chromatin accessibility at non-targeted peaks), then the TF is classified as a putative activator; if it is more negative than the correlation computed in the background, then the TF is classified as a putative repressor; and if the correlation is indistinguishable from that in the background, it is classified as undetermined.

Undetermined TFs include TFs with low expression variation between the two compared conditions, TFs where the low mRNA expression does not reflect the high protein translation rate and proteomic abundance, and bifunctional TFs where a given TF can act as both activator and repressor in the same cell.

Some TFs are classified as putative activators or repressors within specific developmental stages and are undetermined in others. In such a case, some pioneer factors may have higher TF binding activity in iPSCs vs. RPE progenitors, which may become progressively depleted/exhausted or functionally dispensable as differentiation towards RPE progresses, or, presumably, they may exert a dual regulatory role both as putative activators and repressors on select genes and within specific developmental frames. Some TFs are constitutive activators of gene expression across stages, such as CRX and E2F4, whereas others are constitutive repressors, such as EGR1 and ARI3A.

The predicted pioneer factors with the highest classification stringency from pairwise comparison of developmental time points (q-value 0.001) were subject to Gene Ontology enrichment analysis on Panther (GO Ontology database DOI: 10.5281/zenodo.10536401, Released 17 January 2024; Reactome version 85, Released 25 May 2023), ranked based on significance by Fisher’s exact test and corrected by FDR (Table S3).

#### Limitations of the Study

Predicted pioneer and regulatory TFs are not functionally tested in this work.

Targeted biochemical methods have been developed to assess and quantify protein-DNA and protein-nucleosome binding affinity so as to unequivocally identify pioneer factors, ranging from comparative, semi-quantitative in vitro nucleosome reconstitution and binding assays to FRET and large, unbiased SILAC-based nucleosome affinity purification combined with quantitative mass spectrometry [87,88,89,90].

The TF DiffTF predictions presented here may complement existing biochemical and proteomic methods for the empirical validation of pioneer factors in the RPE developmental context.

## Data Availability

C.Z. implemented her research with no contribution from lab affiliates and is the exclusive IP owner, having secured her rights on usage and redistribution of her results (Disclosure of Invention, September 2022), as per art. 39, 40 of legislation EU 2021/695 of the European Parliament and Council of 28 April 2021. For inquiries and collaborations, requests will be considered in light of any intellectual property or confidentiality obligations, and data will be de-identified and shared upon agreement.

## Supplementary Materials

The supporting information can be downloaded at: https://www.mdpi.com/article/10.3390/ijms26125817/s1.

## Appendix A

*Deregulation of predicted pioneer factors binding in pathological epithelial-mesenchymal transitions (EMT).*

Previous work had shown transcriptional similarities between de-differentiating RPE and a metastatic model of cancer [42] by transcriptional profiling of human stem cell–derived RPE induced to EMT by enzymatic dissociation or TGFβ/TNFα treatment, leading to the identification of EMT markers in the RPE.

Epithelial-related markers such as *MITF*, *PMEL17*, *BEST1*, *RPE65*, *RLBP1*, and *TYR* were downregulated upon enzymatic dissociation, and treatment with TGFβ or TNFα elicited similar fluctuations in gene expression.

Recurring genes were deregulated upon both enzymatic and TGFβ/TNFα injury in hRPE-EMT models and in a gland tumor-associated model, affecting development, cell migration, focal adhesion, and integrin complex formation: *ZEB1*, *FOSL*, *FOSB*, *JUNB*, *NFKB1*, *HIC1*, *SMAD3*, *FOXC2*, *LMCD1*, *ELF4*, and *IRF9* mRNAs were upregulated, whereas the RPE factors *SOX4* and *SOX10* mRNAs were downregulated [42].

Upon EMT, also *BMP7*, *FGFR2*, *GNB3*, *GNG11*, *PRKCQ*, *NTNG1*, *ABLIM1*, *UNC5D*, *PLXNB1*, and *ADAMTS1* mRNAs were downregulated, as well as semaphorin genes (*SEMA3A*, *SEMA3D*, *SEMA4A*, and *SEMA6D*) that are known to inhibit axon growth cones.

Instead, interactors of the CasL family members (*MICAL1*, *MICAL2*, and *MICAL3*), involved in axonal growth cone repulsion and actin cytoskeleton reorganization factors (*EPHB1*, *EPHB2*, *EFNB2*, *ITGA2*, *ITGA5*, *PDGFB*, *NGEF*, *ADAM19*, and *RGS3*), were significantly upregulated.

Notably, *SEMA3A*, *SEMA3D*, *SEMA4A*, *SEMA6D*, *ITGA2*, *ITGA5*, *PDGFB*, *NGEF*, and *ADAM19* mRNAs were altered in both enzymatic and TGFβ/TNFα EMT models.

Hepatic fibrosis/retinal tissue fibrosis (*COL8A2*, *MMP2*, *LAMA1*, *IGFBP5*, *PDGF*, *MYH8*, *TGFBR2*, and *SERPINE1* mRNAs), the IL8 pathway, the integrin-like pathway, and the reorganization of actin cytoskeleton architecture were also involved over the course of EMT progression [42].

Inference of upstream regulators of gene expression within promoter regions has revealed that many EMT-induced molecules are likely activated by TFs involved in cancer-associated EMT progression, such as NFKB1, STAT3, RELA, JUN, and HIF1A. EMT downregulated genes were, instead, likely targeted and inhibited by TFs involved in RPE development (OTX2, BCL6, ZFP36) and by kinases that may modulate the RPE-EMT response, such as AKT1, MAPK1, IKBKB, MAP2K1, and MEK1 [42].

Here, we found that many noticeable examples of EMT-induced TFs had a predicted pioneer function in the RPE developmental context, and we identified several related genetic targets by footprinting analysis of extended regulatory regions.

We found that EMT-induced TFs JUN and JUNB activate gene expression in RPE progenitors compared to IPSCs, as well as differentiated RPE compared to iPSCs, along with STAT3; we classify TFs HMGA1 and ATF4, upregulated in EMT, as predicted repressors of gene expression in differentiated RPE compared to RPE progenitors, and SOX7, also upregulated in EMT models [42], as a repressor of gene expression in RPE progenitors compared to RPE (Figure 5D; *all.summary.xls).

FOSL2 and FOSB (upregulated in early response EMT) and SOX10 and OTX2 (downregulated in early response EMT) [42] activate gene expression in differentiated RPE compared to iPSCs (*all.summary.xls). CEBPG, upregulated upon TGFβ/TNFα treatment, represses gene expression in differentiated RPE compared to RPE progenitors. PBX1, downregulated in human EMT models, represses gene expression in RPE progenitors compared to iPSCs (*all.summary.xls).

Among the predicted pioneer factors in human RPE differentiation, *MYC* mRNA is known to be upregulated in both enzymatic dissociation and TGFβ/TNFα EMT-induced human RPE models [42]. MYC represses gene expression in RPE progenitors compared to IPSCs while activating it in RPE progenitors compared to differentiated RPE, possibly enabling progressive transition from iPSCs to neuroepithelial, then RPE differentiated stages (Figure 5D; * all.summary.xls).

We predicted additional pioneer factors in the RPE development, such as JUNB, E2F1, EGR1, EPAS1, SMAD4, ETV5, SP1, E2F1, ETS1, DDIT3, GLI1, JUN, JUNB, STAT3, STAT1, SMAD3, KLF6, ARNT2, SOX1, and ATF4 (*all.summary.xls). These had all been previously inferred as upstream regulators from pathway analysis of differentially expressed (induced) target genes upon TGFβ/TNFα-mediated EMT [42]. Specifically, SP1, STAT3, STAT1, and OTX2 were inferred from pathway analysis of statistically induced genes under enzymatic dissociation.

Instead, candidate pioneer factors MYCN, MEOX2, NRF1, and GFI1 (*all.summary.xls) had been inferred as upstream regulators of statistically downregulated EMT genes upon both enzymatic dissociation-induced EMT and TGFβ/TNFα-induced EMT [49].

As we carried out footprinting analysis in the iRPE, we found that several TFs involved in the EMT process do, in fact, display binding sites within predicted regulatory regions of known EMT targets. ARNT2, for example, was footprinted in extended regulatory domains of targets *ANGPT2*, *NGEF*, *EGFR*, *ITGB3BP*, *LCP1*, *PDLIM4*, *MCM*, and *VEGFA.* We footprinted ATF4 in several predicted domains of the known EMT targets: *ATF3*, *DDIT4*, *JAG1*, *SLC7A1*, *TNFRSF10B*, *CEBPG*, and *VEGFA* (7 FTPs). DDIT3 binding was detected on the following targets: *ATF3*, *CXCL8*, *IL6*, *PPARGC1A*, *TENM4*, and *XBP1*.

For EGR1 we could predict binding onto the known targets: *CCL2*, *EGR2*, *HBEGF*, *NFKB1*, *PLAU*, *PTGES*, *VEGFA*, *CXCL8*, *EGFR*, *LDLR*, *PTGS2*, *SEPRINE1*, *GADD45B*, *SGK1*, and *MYC*. Footprints for JUN were also identified on the following: *ANXA1*, *CXCL8*, *DUSP6*, *EGFR*, *EZR*, *ATF3*, *CDK1*, *FRZB*, *ITGB8*, *CCL2*, *IGF2R*, *IL6*, *SEPRINE1*, *SLC7A1*, *LIF*, *LTBP1*, *MAF*, *PTPRD*, *SGK1*, *TXN*, *WNT5A*, *MCF2L*, *MYC*, *PTPN1*, *PLAU*, *PTGS2*, *SDC1*, *SMAD7*, and *VEGFA*. For JUNB, binding was also identified on predicted regulatory regions of several known EMT targets: *ATF3*, *DUSP1*, *IL6*, *PLAU*, *ID2*, *SERPINE1*, and *SGK1*. For MEOX2, we could identify the following footprinted targets: *CDKN1C*, *SLC44A3*, *HBEGF*, and *TNFAIP3*.

SMAD3 binding was detected onto the following EMT targets: *CCL2*, *COL3A1*, *FST*, *HBEGF*, *MMP1*, *PDGFB*, *PTP4A3*, *GADD45B*, *IL6*, *PPARGC1A*, *SMAD7*, *ID2*, *IL11*, *IL1B*, *JAG1*, *SERPINE1*, *TGFB1*, *PPARD*, *MYC*, and *VEGFA*.

SP1 binding was footprinted onto the following known EMT targets: *AKAP12*, *IL4R*, *IRF1*, *PLAU*, *PLAUR*, *ATF3*, *HMGA1*, *KRT19* (8 FTPs), *PIM1*, *TNFRSF10B*, *BMP7*, *MYC*, *TNFAIP3*, *DLC1*, *EZR*, *HBEGF*, *KRT19*, *NES*, *SERPINE1*, and *TGFB1. STAT3* binding was detected for the following EMT targets: *ALDH1A1*, *CXCL12*, *RPE65*, *COL3A1*, *SGK1*, *FLRT3*, *TRPM3*, *VCAN*, *WNT5A*, *MITF*, *PTPN13*, *SOX6*, and *VEGFA*.

Among the OTX2 presumptive targets from previous IPA pathway analysis [42], we footprinted (IPSCsvsRPE) *EFNA5* as well as *ENPP2* and *MITF*. Among those EMT differentially expressed genes inferentially bound by SMAD3, we footprinted *BCL3*, as well as *TGFB1* and *PTP4A3* (Table S4). OTX2 binding was detected in extended regulatory regions of the following EMT targets: *DCT*, *EFNA5*, *MITF*, *TYR*, *PTPRZ*, and *TRPM1*.

KLF5, acutely induced upon enzymatic dissociation of EMT [42], is a predicted repressor of gene expression in the RPE compared to hiPSCs (Figure 5D; *all.summary.xls). Moreover, the *KLF5* regulatory region was footprinted by several predicted pioneer TFs, such as FOXP1, OTX2, RARB, PITX1, and TWIST1.

MITF was footprinted onto extended regulatory regions of the following targets (IPS vs. RPE): *ATP1A1*, *TRPM1*, *SLC7A8*, *GPNMB*, *INPP4B*, *OTX2*, *DCT*, *EDNRB*, *STXBP1*, *ITGB8*, *SLC7A8*, *PPM1H*, *SDC1*, *TRPM1*, and *TYR*. Examples for NFKB1 targets include BCL3, *CRMP1*, *CXCL8*, *DUSP1*, *EGFR*, *MYC*, *CCL2*, *ENPP2*, *IL1B*, *IL6*, *IRF1*, *JAG1*, *NFKB1*, and *PLAU*. BCL6, MITF, and SPDEF were also inferred upstream regulators of EMT (downregulated) genes, whereas NFKB1, SMAD2, TP63.0, TP63.1, and HIF1a had been associated with EMT upregulated ones [42].

The EMT may reactivate cell cycle genes and early developmental pathways normally suppressed in the RPE specification context, as well as latent, alternate cell fates. *DBP*, *FOXJ1*, and *HLF* expression drop rapidly as soon as the EMT process is initiated, suggesting that the genetic repertoire of “early response TF genes” could be therapeutically targeted first and regulatory “late response TF genes” after, such as *EGR3*, *HIC1*, *FOSB*, and *SOX17*, which are induced at later stages of disease progression.
