# Supplementary material for: Molecular Determinants of the Human Retinal Pigment Epithelium Cell Fate and Potential Pharmacogenomic Targets for Precision Medicine
Source: Int J Mol Sci. 2025 Jun 17;26(12):5817. doi: 10.3390/ijms26125817 (PMC12192794; doi:10.3390/ijms26125817)
Supplement: Supplementary file 1 [file ijms-26-05817-s001.zip › ijms-3599407-supplementary.pdf]

International Journal of
Molecular Sciences

MDPI

Article

Molecular Determinants of the Human Retinal Pigment Epithelium Cell Fate and Potential Pharmacogenomic Targets for Precision Medicine.

Cristina Zibetti [1,*]

Supplementary Figures

Figure S1

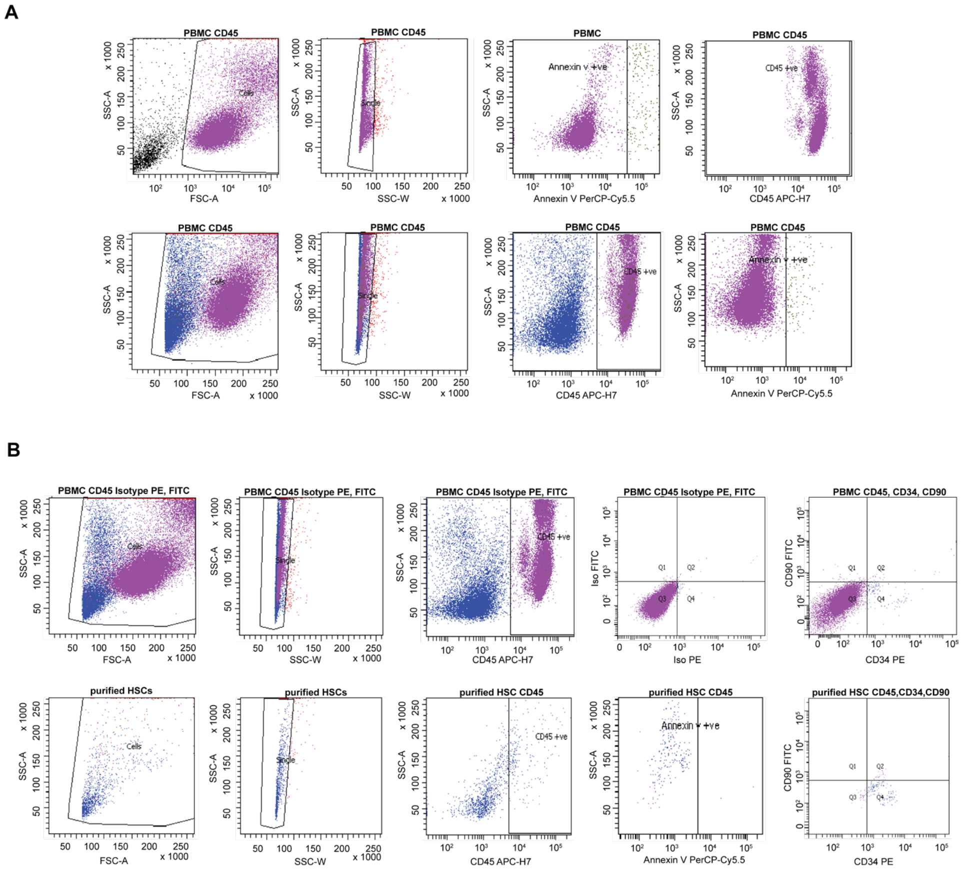

Supplementary Figure S1. Peripheral blood mononuclear cells (PBMCs). **A)** PBMCs were extracted, stained with CD45+(APC-H7) antibody and analyzed by flow cytometry for annexin V-PerCP-Cy5.5 binding to the cell surface to detect apoptotic and necrotic cells. Between 97.3% and 99.4% of extracted PBMCs recovered across trials were viable, based on Annexin V exclusion (2.7% PBMC and 0.6%. CD45+ve cells are positive for Annexin V-PerCP Cy5.5 from two donors). **B)** Of all PBMCs that stain for CD45(APC-H7)+ve, between 0.2% and 0.5% are also positive for CD34+(PE) following immunomagnetic purification (differences were observed across donor-derived batches), and 0.1% were found to be double positive for CD34+(PE) and CD90+(FITC), compared to fluorochrome-matched isotype controls, counterstaining the most proliferative CD34+ HSCs subfraction. Overall, the efficiency of retention for CD34+ HSCs from PBMC is around 83.9%, of which 13.7% are double positive for CD34+ and CD90+.

Figure S2

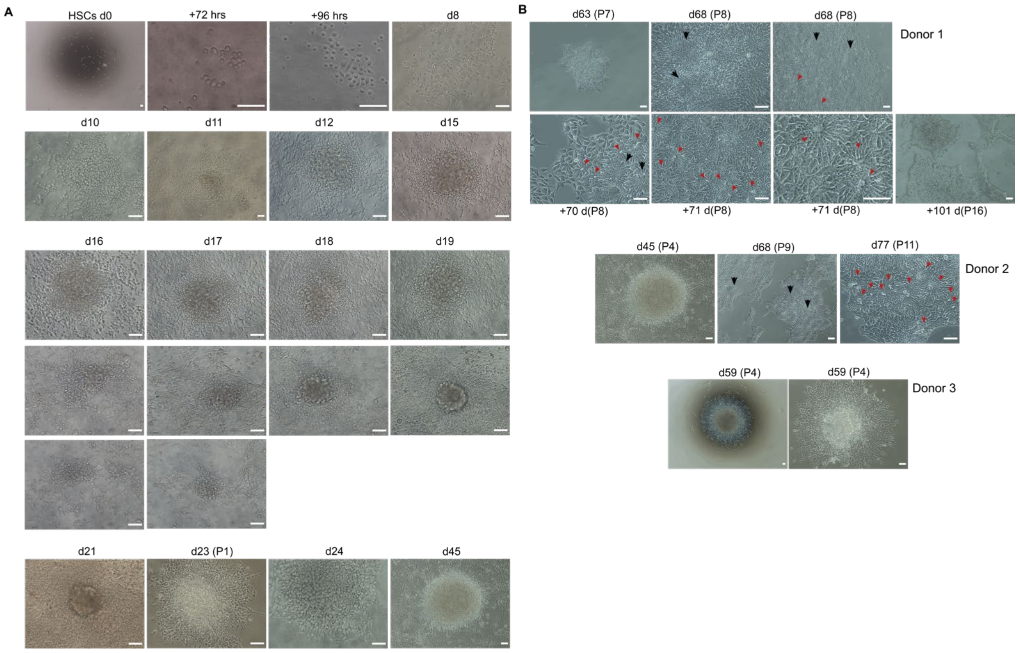

Supplementary Figure S2. Reprogrammed HSC CD34+ **A)** Time-course imaging of reprogrammed HSC CD34+ cells showing pre-iPSCs colonies emerging during the first three weeks and after the first passage. Images acquired in bright-field (4x,10x,20x,40x) and phase contrast (10x,20x,40x) with Nikon TS100. Scale bar is 50 μm. **B)** Representative bright field images displaying enriched CD34+ HSC cell eluates were acquired, as well as embryoid bodies. Presumptive iPSCs clones obtained upon reprogramming and derivative cell lines were monitored across passages for up to 6 months in culture. Cells were tested once to exclude mycoplasma contamination. GFP incorporation was ascertained by nucleofection, and the emergence of primary clones from reprogrammed cells was monitored over time by colony expansion and proliferating iPSCs were maintained and tested.

Figure S3

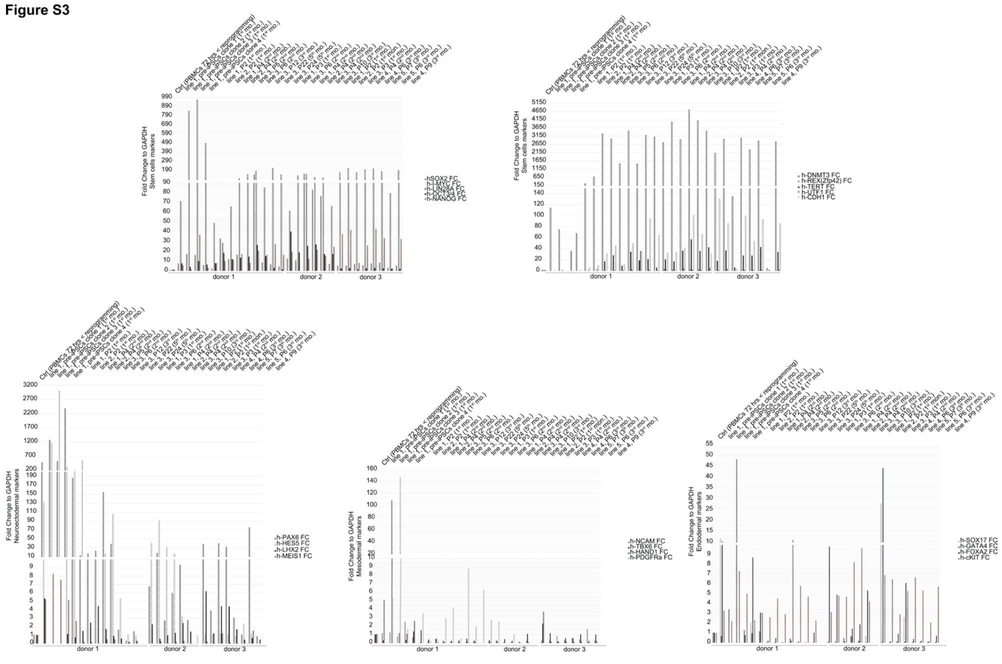

Supplementary Figure S3. Additional RT-qPCR are shown from iPSC clones and derivative cell lines across passages (P) displaying variations in neuroectodermal, mesodermal and stemness markers over time, including *PAX6, HES5, LHX2, MEIS1, NCAM, TBX-6,*

HAND1, *PGFR-a*, *SOX17*, *GATA-4*, and *FOXA2*. Statistically significant variations in gene expression are featured in Figure 1C, 2D, and 3D and E.

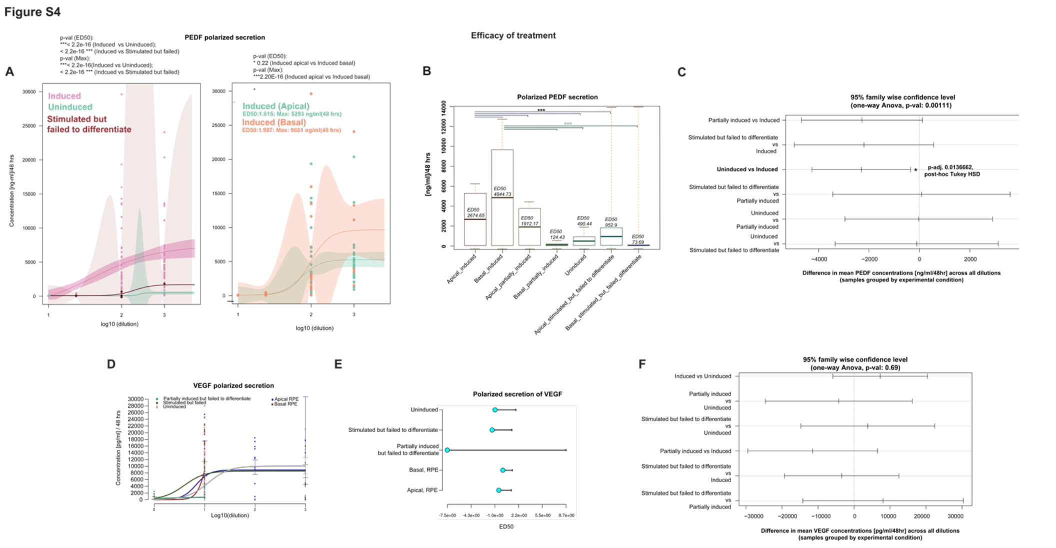

Supplementary Figure S4. Efficacy of PEDF polarized secretion by model fitted 4PL log logistic. A) Dose-response curve features PEDF concentration [ng/ml]/48 hrs by log10(dilution). *p*-values were calculated on concentrations estimated at ED50 and points of maximum growth across dilutions. Estimated concentration at maximum growth (Max): PEDF Apical (5293); PEDF Basal (9661). Uninduced cells secrete significantly lower PEDF than fully formed RPE cells and do not phenotypically display signs of induction, such as a stereotypically cuboidal, hexagonal-shaped epithelial monolayer, nor melanogenesis although sporadic pigmented foci can be detected. Partially induced ones are donor-matched and display pigmented patches although they fail to coordinate into a pigmented monolayer. Stimulated ones derive from different donors subject to the same treatment. **B)** PEDF secretion across experimental conditions and donors. Model fitted: 4PL log-logistic. Concentrations estimated at ED50 are reported. *p*-values on concentrations estimated at PMG (estimated points of maximum growth) (bilateral t-student test; ∣Stat-t∣ ≥ T $\alpha/2$); error bars as 0.95 C.I. Apical induced vs Basal induced > 0.05; Basal induced vs Basal partially differentiated < 0.05; Basal induced vs Basal stimulated but failed to differentiate < 0.001; Uninduced vs Basal stimulated but failed to differentiate < 0.001. Concentrations [ng/ml]/48 hrs estimated at ED50: Apical induced (2674.65); Basal induced (4844.73); Apical, partially induced (1912.17); Basal, partially induced (124.43); Uninduced (490.44); Apical, stimulated but failed to differentiate (952.9); Basal, stimulated but failed to differentiate (73.69). **C)** 95% family-wise confidence level (one-way Anova, p-val: 0.00111). Difference in mean PEDF concentrations [ng/ml/48hr] across all dilutions (samples grouped by experimental condition). Induced versus uninduced: p-adj. 0.0136662,* post-hoc Tukey HSD. Efficacy of VEGF polarized secretion by NPRL. **D)** VEGF concentration [pg/ml]/48 hrs by log10(dilution). N-parameter weighted logistic regression curve fit (NPLR) (weight method: residuals). Npar: w-3PL (apical), w-3PL (basal); w-5PL (partially induced but failed to differentiate); w-3PL (stimulated but failed), w-3PL (uninduced). Concentration [pg/ml] / 48hrs was estimated at point of maximum growth (ED50): VEGF Apical (4436.38); VEGF Basal (10839.64); partially induced but failed to differentiate (1574.37); stimulated but failed (4281); uninduced (5024). **E)** Uninduced epithelial cells still secrete VEGF, although the mesenchymal-to-epithelial transition is incomplete. 0.95 C.I. (ED50): RPE Apical ($3.31 \times 10^{-1} – 2 \times 10^{1}$); RPE Basal ($1.17 \times 10^{0} –2.43 \times 10^{1}$); Partially induced but failed to differentiate ($2.96\times 10^{-8} – 8.3 \times 10^{8}$); Stimulated but failed to differentiate

(4.07 × 10^{-2}– 2.08 × 10^{1}); Uninduced (1 × 10^{-1} – 6.03 × 10^{1}). **F)** 95% family-wise confidence level (one-way Anova, p-val: 0.69). Difference in mean VEGF concentrations [pg/ml/48hr] across all dilutions (samples grouped by experimental condition).

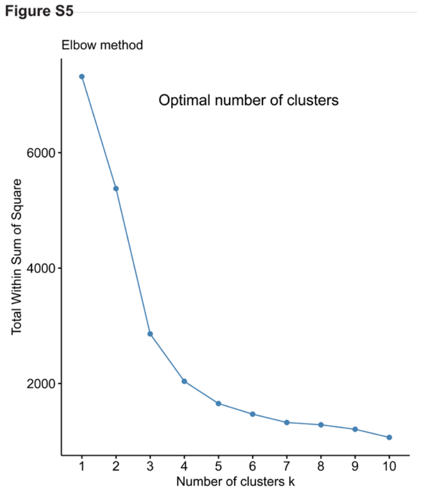

Supplementary Figure S5. Assessment of the optimal number of k-clusters. Assessment was carried out for k-means clustering of coding mRNAs (Figure 3E) by the elbow method.

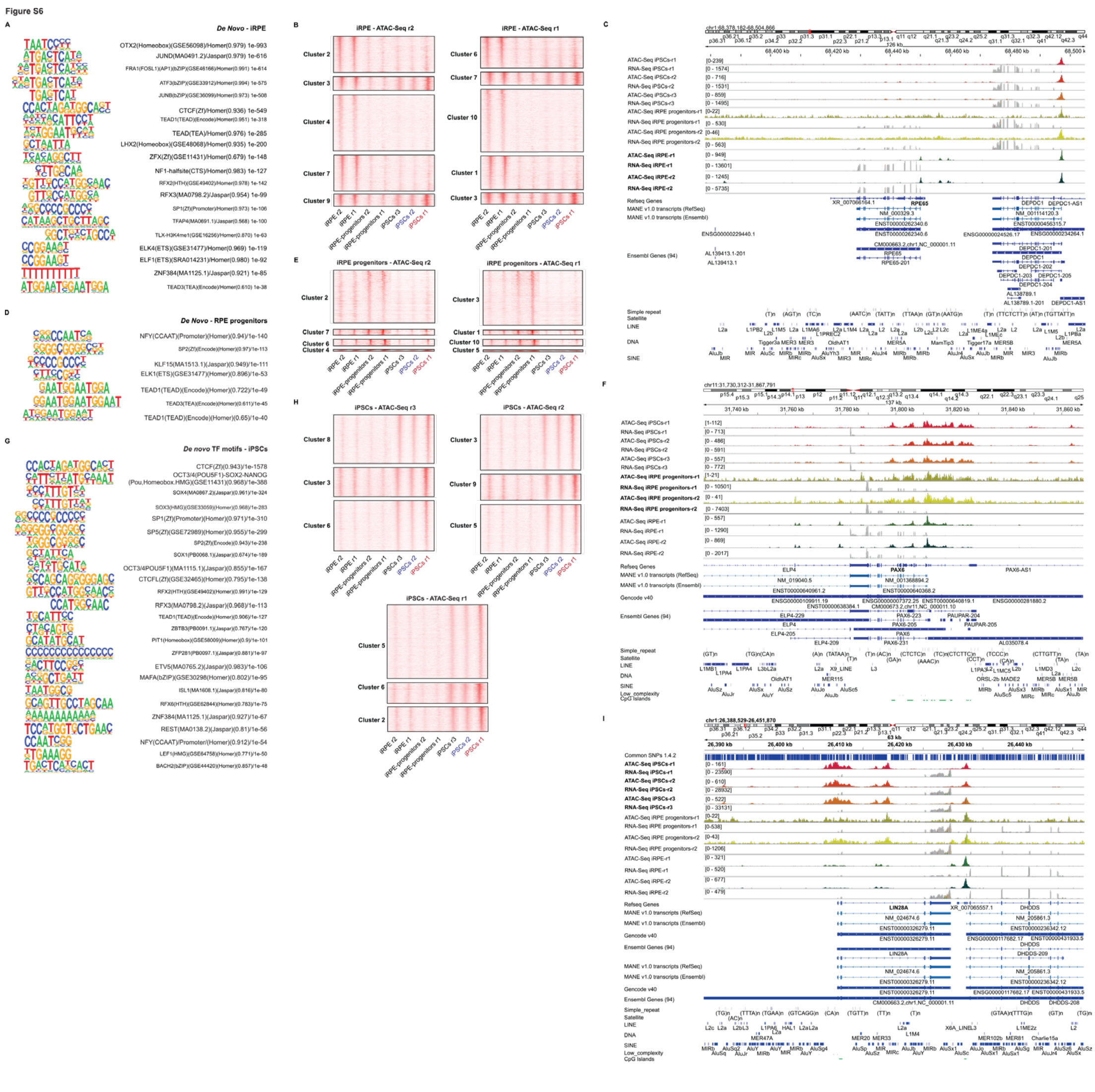

Supplementary Figure S6. Open chromatin in human RPE development. (A,D,G) De Novo Motif enrichment. DNAse peaks for motif enrichment analysis were called (see method 4.4) reflecting chromatin regions with interspersed nucleosome gaps in iRPE (A), RPE progenitors (D) and hiPSCs(G). Peaks from experimental replicates were merged by experimental condition and scanned for motif enrichment. Motif enrichment was carried out by binomial scoring of novel oligonucleotides and comparison against known motifs, including the Jaspar 2022 nonredundant core motifs collection [78]. TF logos are displayed, together with Pearson's correlation reflecting similarity to known deposited PWMs. Motif enrichment was carried out on repeats masked hg38 genome (GRCh38.p13) at an empirically determined length (len 8-12), with top 10 instances optimized. PWMs of TFs with detectable stage-matched expression by RNA-Seq are indicated in higher font. Indentation signifies grouping by motif similarity. **(B,E,H)** Open chromatin clusters in human RPE development. Genome-wide reads histograms across nucleosome-free regions were subject to K-means clustering (10 Ks, 100 iterations set) in iRPE (B), RPE progenitors (E) and hiPSCs(H). **(C,F,I)** IGV custom tracks. IGV custom tracks were configured on the human genome GRCh38 and auto-scaled featuring progressive changes in chromatin accessibility and corresponding gene expression at target genes *RPE65* (C), *PAX6* (F) and *LIN28* (I).

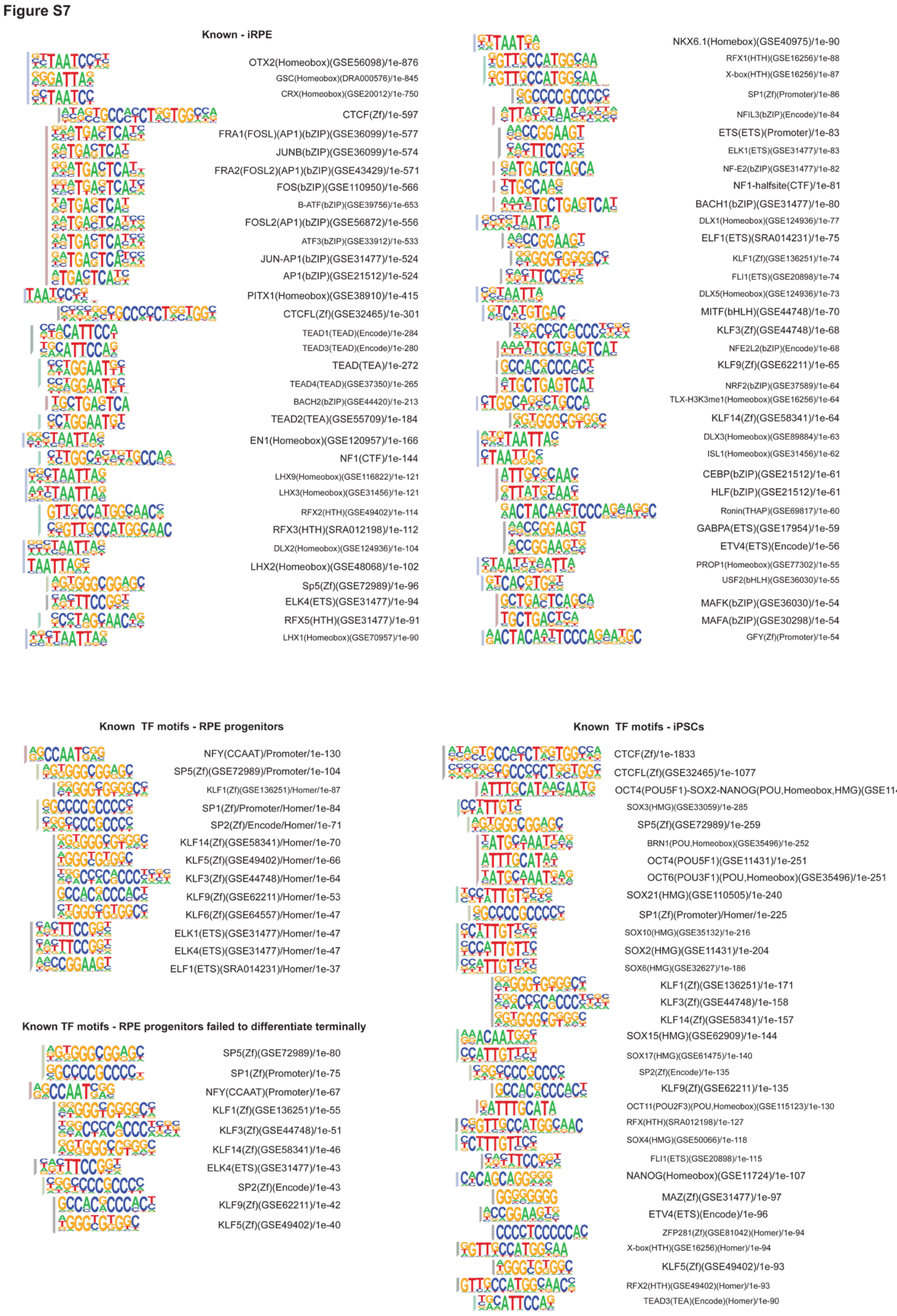

Supplementary Figure S7. Known motif enrichment. DNAse peaks were identified, reflecting accessible (open) chromatin regions with interspersed nucleosome gaps (see method 4.4). Peaks of open chromatin from experimental replicates were merged by experimental condition and scanned for motif enrichment. Known motif enrichment was carried out by binomial scoring of deposited PWMs (position weight matrices) and TF logos are displayed. Indentation signifies grouping by motif similarity. Motif enrichment was carried out on repeats masked hg38 genome (GRCh38.p13) at an empirically determined length (len 8-15), with top 10 instances optimized. PWMs of TFs with detectable stage-matched expression by RNA-Seq are indicated in higher font. In smaller font are enriched PWMs likely reflecting variations of the same motif instances.

Figure S8

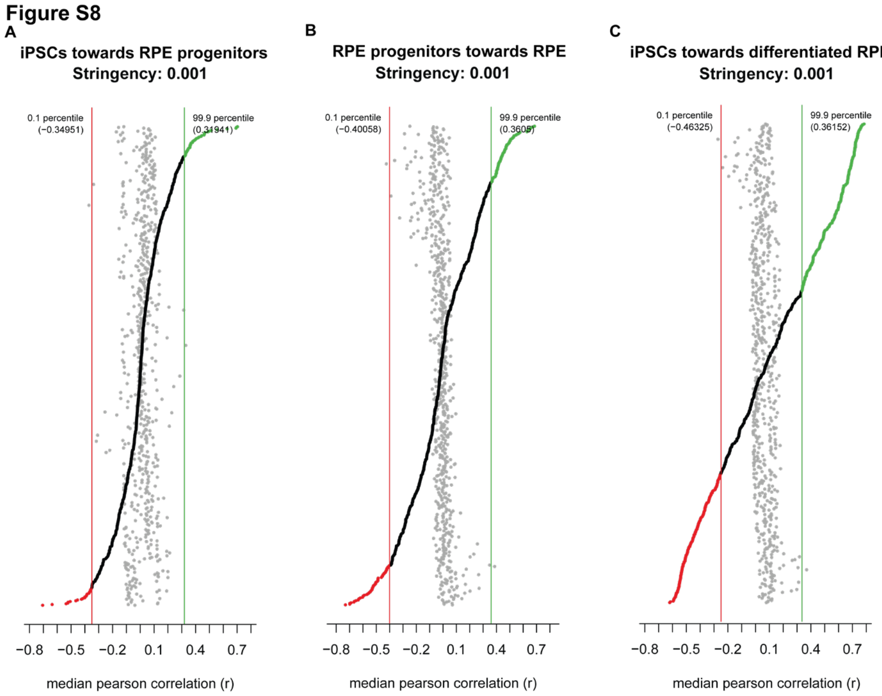

Supplementary Figure S8. TF expression levels are correlated with chromatin accessibility of its own target sites. Median Pearson's correlations for all TFs, from bottom to top (most significant on the y axis, past the threshold). TF activity is measured in IPSCs versus RPE progenitors (A), RPE progenitors versus iRPE (B) and iPSCs versus iRPE (C). For every TF, TF expression levels (x) are correlated with the chromatin accessibility of TF-specific target sites compared to non-targeted peaks (peaks bound by unrelated TFs) (y). If the correlation between the expression level of any given TF and the chromatin accessibility of its own target peaks (foreground) is more positive than the correlation computed in the background distribution (chromatin accessibility at non-targeted peaks), then the TF is classified as a putative activator; if it is more negative than the correlation computed in the background, then it is classified as a putative repressor, and if the correlation is indistinguishable from that in the background, it is classified as undetermined. Each dot is a TF. Red is repressor, black is undetermined and green is activator. The more the two vertical lines of threshold significance move towards the outside, the larger the undetermined area, and the more significant and stringent the call for those TFs that are activators or repressors. The values indicated denote the particular percentiles of the background distribution across all TF as a threshold for activators and repressors and are used to distinguish real correlations from noise (i.e., activator/repressor from undetermined).

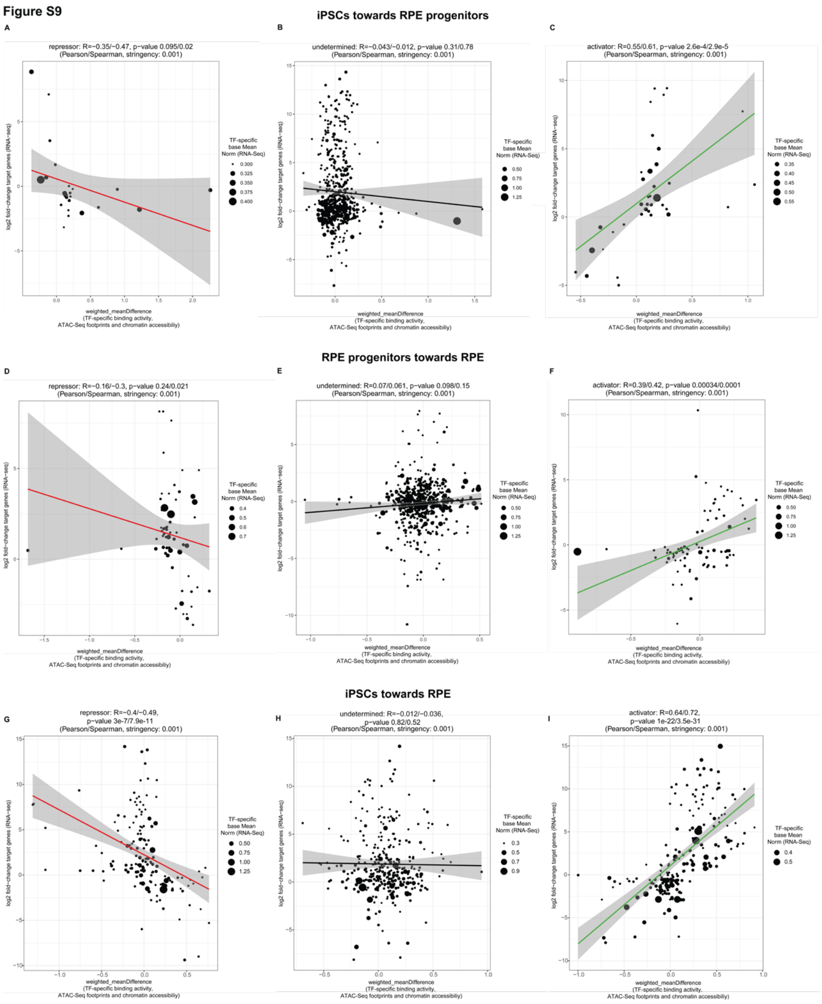

Supplementary Figure S9. Weighted mean difference as differential TF activity between experimental conditions. Differential TF activity based on detectable FTPs (TFBS) and chromatin accessibility of target genes by ATAC-Seq, plotted against corresponding variations in target gene expression. Differential TFs activity between conditions is referred to as the weighted mean difference across GC bins between the experimental conditions being compared (X axis) and plotted against the corresponding log2 fold change variation in target gene expression. Differential TF activity is measured in IPSCs versus RPE progenitors (A,B,C), RPE progenitors versus iRPE (D,E,F) and iPSCs versus iRPE (G,H,I). The difference in chromatin accessibility around TF motif centers is calculated between foreground chromatin accessibility (TF-specific TFBS) and background distribution at non-target sites (unrelated TFBS). High TF activity resulting in downregulation of genes is represented by the regression line with a negative slope (red), signifying repressors. Hence, the lower the TF binding, the higher the repression release of target genes. Conversely, for putative activators, the higher the recruitment on chromatin regions, the higher the derived expression of target genes, and the lower the TF activity, the lower the expression of target genes.

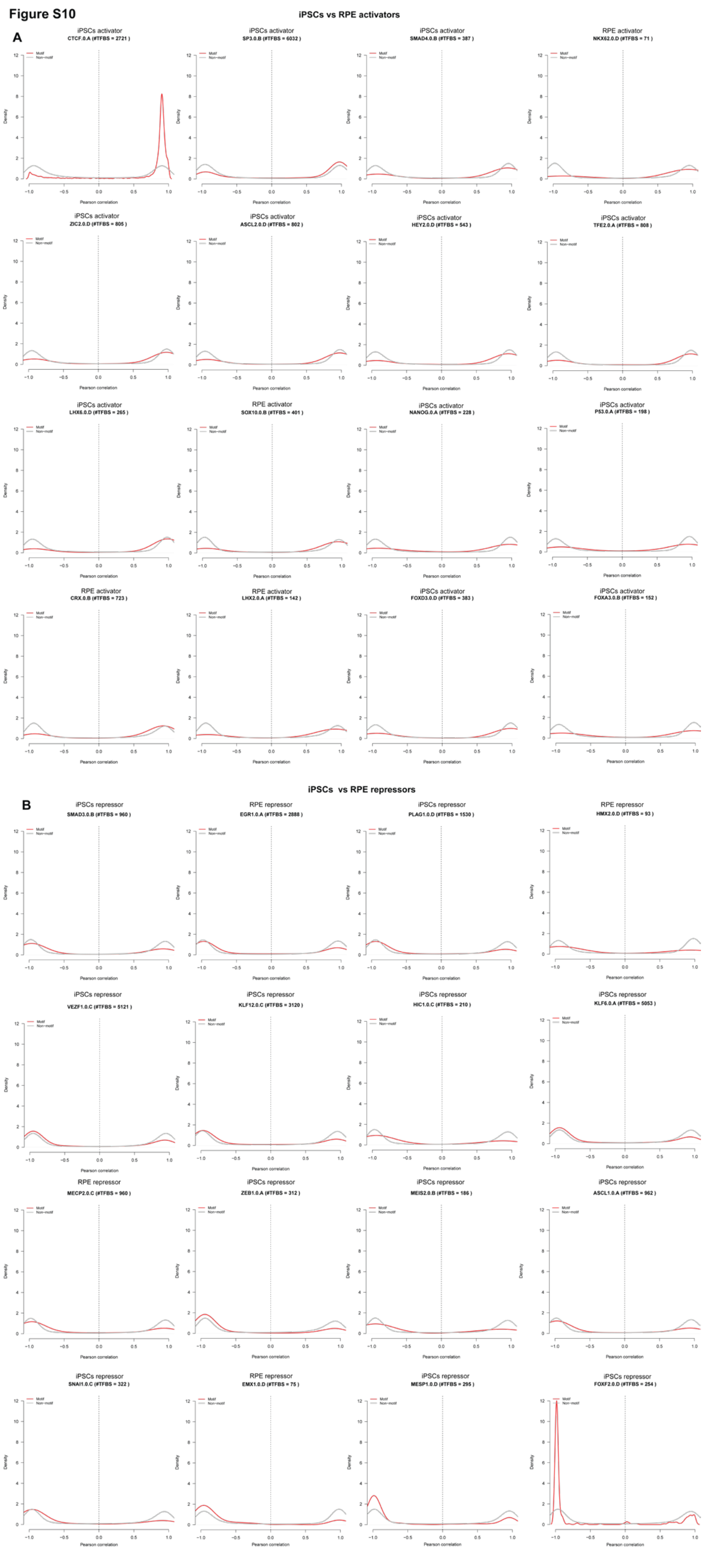

Supplementary Figure S10. Extracted read densities around TF-specific motifs by TF gene expression. Select TFs that are classified as putative activators / repressors / undetermined in function are displayed, regardless of their directionality (differential activity by stage, as in Figure 5 and *.summary.xls files). Predicted activators in iPSCs versus iRPE are displayed in (A) and predicted repressors in (B). Pearson's correlation is computed between TF expression level and chromatin accessibility (reads density distribution around motif center) on the foreground set of TF-specific peaks (red) and background set

(grey) of peaks bound by unrelated TFs. Chromatin accessibility is computed as log2 fold-change in read density from all putative predicted TF binding sites for the indicated TF, only to TF-specific bound sites (FTPs). If the highest Pearson's correlation in the foreground (motif) is higher than in the background (non-motif), then the TF is classified as an activator. If it is lower, the TF is classified as repressor. The top panel features some of the activators that induce chromatin accessibility in the transition from iPSCs to RPE, as in Figure 4. TFs that are most stringently classified (smallest adjusted p-value, classification stringency at q=0.001) are displayed. The bottom panel features cases of repressors that mediate chromatin compaction during the transition from iPSCs to RPE. The extensive list of all classified TF activators and repressors is reported in "iPSCsvsRPE.all.summary.xls" sorted by alphabetical order where "undetermined" includes unexpressed or bifunctional TFs. LHX2 is classified as as a repressor (IPSCsvsInt) (weighted mean difference, wdf < 0) indicating higher regulatory activity as repressor in IPS compared to RPE progenitors (qval < 0.05), as an activator (IPCsvsRPE) and preferentially active in RPE compared to IPSCs (wdf > 0), and as an activator (qval < 0.01) (IntvsRPE, wdf > 0) signifying higher activity in RPE compared to RPE progenitors.

Figure S11

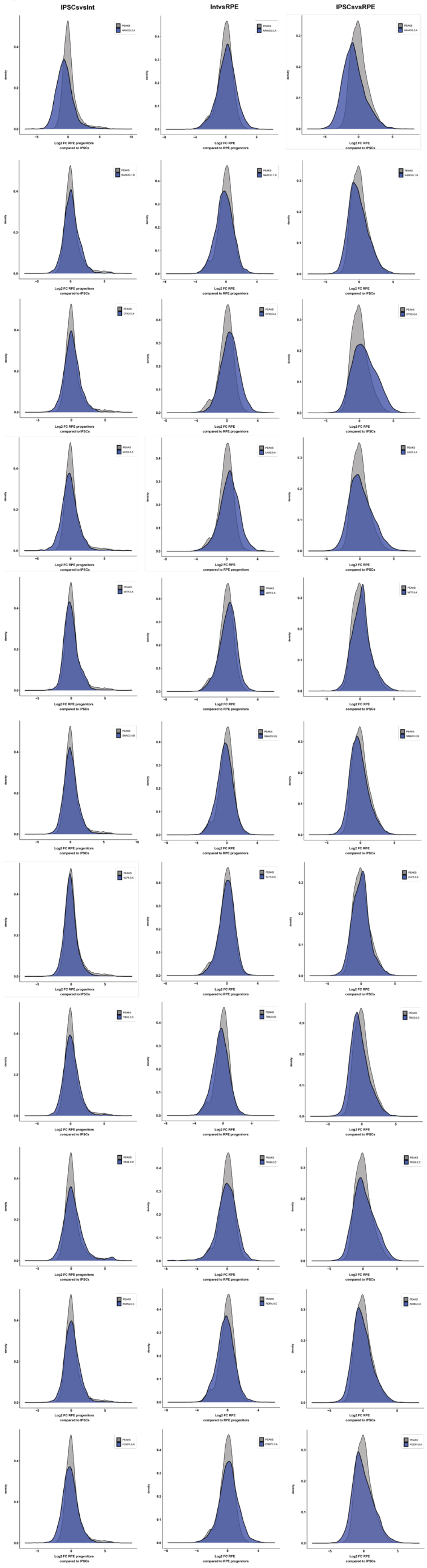

Supplementary Figure S11. Representative diagnostic plots for differential accessibility of TFBS during RPE development, as shift in regulatory function. Density plots featuring

transitions in chromatin accessibility of TFBS from hiPSCs to RPE progenitors to terminally differentiated RPE. Pairwise comparison of read density across TF motif centers is computed between the indicated developmental time points. Log2 fold-change in read density between time points is displayed for all peaks independent of any TF (background, grey) and for TF-specific bound peaks (FTPs) (foreground, purple). Directionality signifies differential TF activity by stage, regardless of the classification as activator / repressor / undetermined. The order of the two conditions (*.summary.xls) matters. The condition specified first is the reference condition. For the "IPSCvsRPE" files, for example, all log2 fold-changes will be the log2fc of RPE as compared to IPSC. A positive log2 fold-change means it is higher in RPE as compared to IPSCs. Whether the TF activity is detected as an activator or repressor, or undetermined, is reported in *.summary.xls files (weighted mean difference).

Excel Tables

Table S1_ Oncogene exome analysis of hiPSCs vs. donors' PBMCs.

Donors' PBMCs and derivative iPSC cell lines at passage 10 were subject to whole exome sequencing. All libraries were subject to QC, as indicated in methods 4.3.
GATK (v4.0) was used to call single-nucleotide polymorphisms (SNPs) as well as Insertions/Deletions (InDels), and ANNOVAR (2015Dec14) to annotate variants [74].
Missense SNVs, synonymous SNVs, frameshift insertions, frameshift deletions, non-frameshift insertions, non-frameshift deletions, frameshift block substitutions, non-framshift block substitutions, stopgains, and stoplosses were identified. Functional distribution for SNVs and InDels is displayed on the first page.

Analysis of PBMCs and derivative hiPSCs was carried out to reveal somatic mutations acquired in the reprogramming process. SNPs and InDels were cross-referenced for oncogenicity against the Catalogue Of Somatic Mutations In Cancer database (COSMIC) and for clinical significance (ClinVAR) regardless of the penetrance or zygosity detected.
SNVs and InDels classified in the Catalogue Of Somatic Mutations In Cancer (COSMIC v70) are reported, regardless of whether they affect exonic regions, splice junctions, upstream and downstream regulatory regions, 5'UTR and 3'UTR or rather intergenic and intronic regions and regardless of the zygosity detected.
SNVs and InDels with known clinical significance (CLNSIG) are also reported and indicated as involved in drug response, risk factors or pathogenesis even if low penetrance or loss of heterozygosity is required as per ACMG/AMP recommendations. The related ClinVar allele ID (CLNALLELEID) and disease name (CLNDN) are also indicated. SNVs and InDels with a known benign or neutral clinical significance are not included.

For every SNV and InDel, gene name and transcript(s) identifiers are reported, together with the sequence change from reference base to alternate allele(s) and, in the case of missense mutation or frameshift mutation, the resulting amino acid change. For instance, a SNP of the gene P53 with missense function of exon 3 on base 98 of the cDNA from c to g (c.C98G) resulting in a protein change on amino-acid 33 from proline to arginine (p.P33R) will be referred to as TP53:NM_001126118:exon3:c.C98G:p.P33R. A structural variant resulting in nucleotide insertion with an amino acid change will be denominated correspondingly. For instance, for ALMS1:NM_015120:exon1:c.74_75insGG:p.E25fs, c.74_75insGG indicates a change in base c at position 74 of the coding sequence, with an insertion (GG) in exon 1, resulting in protein change with insertion of amino-acid 25 as Glutamic acid.
Functional prediction of deleteriousness for exonic, non-synonymous SNVs was assessed by SIFT (D (Deleterious, score ≤0.05), by Polyphen2_HDIV for rare alleles and complex phenotypes or gwas (D = Probably damaging, score ≥ 0.957), by Polyphen2_HVAR for

diagnostics of Mendelian disease (D = Probably damaging, score ≥ 0.909), by MutationTaster (dbNSFP version 3.3a) (A= stop-gain SNVs and Disease_causing_automatic; D= Disease_causing for rare alleles whose frequency information is not available in HapMap), by LRT (D = Deleterious), by MutationAssessor (H= high-risk) and by FATHMM (D = Deleterious, score ≤-1.5). The above are similar and all predict whether an amino acid substitution affects protein function; only coding variants have these annotations. For synonymous and non-synonymous SNVs, conservation score was assessed by phyloP, SiPhy, gerp++ and CADD. Highly scored variants may confer increased susceptibility.

Genotype was inferred based on VAF (variant allele frequency), as percentage of sequence reads (allelic depth) observed matching a specific DNA variant divided by the overall sequencing coverage at that locus. For heterozygous germline variants 50% VAF is expected, for homozygous ones, allele frequency should be near 100%, and reference loci should be near 0%. Deviation from such percentages may reflect bona fide somatic mutations and mosaicism. If the VAF is far higher than expected, it could indicate that the variant is either germline or in a region of loss of heterozygosity. Allele frequency in each sample is indicated next to the Format. GT: genotype, encoded as allele values separated by either of / or ∣.

The allele values are 0 for the reference allele (what is in the Ori_REF field), 1 for the first allele listed in Ori_ALT, 2 for the second allele list in Ori_ALT and so on. 0/0 and 1/1 represent homozygous. 0/1 represents heterozygous. AD: Allelic depths for the ref and alt alleles in the order listed.

Table S2_GO_Terms_Enriched.

Clusters of nucleosome-free, coordinately accessible open chromatin identified by K-means clustering of sequencing reads normalized by library size (Fig. S6, B,E,H) (see method 4.5) were screened for enriched gene ontologies along the three stages of iRPE genesis from pluripotency, through RPE-committed precursors, to terminally differentiated RPE.

---Clusters from merged peaks derivative from experimental replicates were subject to Gene Ontology enrichment. First, assignment of chromatin regions to putatively regulated genes is not limited to the nearest promoter. Instead, chromatin regions are assigned to all promoter-extended regulatory domains that each chromatin region overlaps with, defined by extending TSS regions 5000 bp upstream and 1000 bp downstream for up to 1000000 bp max extension, as described previously. GO enrichment was computed with GREAT [79,80] by binomial scoring and the rank was corrected by multiple Hp testing (Bonferroni-Hochberg). Both mouse and human ontologies are reported, whenever identified. The gene ontologies represented here are functionally and developmentally enriched for the TF related oligonucleotides (de novo motif enrichment) indicated in Fig. S6A,D and G.

Table S3_GeneOntologies_PioneerTFs.

The identified TFs were subject to Gene Ontology enrichment analysis on Panther (GO Ontology database DOI: 10.5281/zenodo.10536401, Released 17 January 2024; Reactome version 85, Released25 May 2023) by Fisher's exact test and corrected by FDR. Displayed results have FDR p-val < 0.05. Functional subclasses are indicated in bold. Parent terms are indented. The pioneer factors reported are the ones identified at the highest stringency (classification_q0.001_final), along with the weighted mean difference reflecting preferential activity in the former (wmd < 0) or latter (wmd > 0) developmental condition reported. The TFs populating select Panther GOs are also listed in the Table.

Table S4_TFBS_FTPs_GeneOntologies.

Gene ontologies functionally enriched and developmentally coordinated by select human TFs are indicated. Chromosomal coordinates within the human genome (TF-specific binding sites) are also reported featuring direct regulatory targets of potential pharmacological relevance.

TF binding sites (footprints) referenced across the text are reported in the first sheet. TFs for which an activating or repressive function on gene expression could be consistently predicted along iRPE development (constitutive activators/repressors), and highly expressed TFs by RNA-Seq, are also displayed with their binding repertoires.

All chromosomal coordinates reported here (200 bp peaks +/- 100 bp from footprint center) reflect empirically detected TFBS by footprinting analysis [16] (featureCounts v1.6.3 from DiffTiff Suite).

Assignment of footprinted peaks to putatively regulated genes (targets) is not limited to the nearest promoter. Instead, footprinted peaks are assigned to all promoter-extended regulatory domains that each footprinted peak overlaps with, with 70% of footprinted peaks assigned to at least 2 genes. All extended promoter domains are defined by extending TSS regions 5000 bp upstream and 1000 bp downstream for up to 1000000 bp max extension in the reference human genome hg38, as described previously [79,80]. GO enrichment was computed with GREAT by binomial scoring of gene hits (extended regulatory domains in the input dataset, i.e., footprinted) and the rank was corrected by multiple Hp testing (Bonferroni-Hochberg).

Relevant gene ontologies co-regulated (footprinted) by the same TF are reported. For every called gene (hit) populating any given GO, multiple regulatory peaks (TF-specific footprinted peaks) may have been identified, in which case, at least two footprinted locations (most proximal to the TSS) are reported, with relevant chromosomal coordinates and distance from TSS. While functional assignment is not experimentally tested, footprinted peaks that are proximal to promoter regions (distance from TSS) are likely to exert a direct regulatory function on the indicated target gene.

Unless otherwise specified, peaks (FTPs) that are consistently bound across at least two out of three RPE differentiation comparisons (hIPSCsvsInt; IntvsRPE; hiPSCsvsInt), are reported here (mergepeaks.pl -d given, Homer v4.11) [77]. Hence, the TFBS chromosomal coordinates reported here do not necessarily reflect stage-specific GO ontologies, only GO ontologies for TFBS that are consistently bound across stages by a given TF (peaks derived from physical merge). All these regions represent high-confidence Transcription factor binding sites within the human genome, since they display TF-specific PWMs, focal depletion of the reads around the ATAC-Seq peak center, indicative of actual binding, and paired variation of chromatin accessibility across stages, resulting in differential TF activity (Fig. 5). Additional ontologies related to stage-specific peaks and bearing stage-specific TF footprints may be reported, if expressly stated. (For a comprehensive list of stage-specific regulatory relevance of each and every given TF in the human RPE epigenome, refer to *.all.summary.xls files and highest stringency classification in Table3). TFBS associated with predicted activators and predicted repressors are displayed on different sheets. TFs displaying high expression level by RNA-Seq are also reported, regardless of predicted activity (activator/vs repressor/vs undetermined). The GOs displayed here represent gene ontologies that are functionally enriched and developmentally coordinated by the TF indicated. Both mouse and human ontologies are reported, whenever identified.

Excel Peak files:

IPSCvsInt.all.peaks.xls, IPSCvsInt.all.summary.xls, IPSCvsInt.all.TF_vs_peak_distribution.xls; IntvsRPE.all.peaks.xls, IntvsRPE.all.summary.xls, IntvsRPE.all.TF_vs_peak_distribution.xls; IPSCvsRPE.all.peaks, IPSCvsRPE.all.summary,
iPSCvsRPE.all.TF_vs_peak_distribution
* stands for developmental pairwise comparison, as indicated above.

***.peaks.xls files:** Permutation, peakID; DESeq_baseMean; l2FC; DESeq_ldcSE; DESeq_stat; *p*-val; *p*-val_adj. Exactly two conditions have to be specified. The order of the two conditions matters [16].
The condition specified first is the reference condition. For the "IPSCvsRPE" example, all log2 fold-changes will be the log2fc of RPE as compared to IPSC. That means that a positive log2 fold-change means it is higher in RPE as compared to IPSCs. Consequently, the final TF activity (denoted as the weighted mean difference in the *summary.xls output tables) will have the same directionality. Hence, for positive values of weighted mean difference (*summary.xls), the TF is preferentially active in the latter condition being mentioned (i.e. RPE in the IPCSvsRPE.summary.xls file), whereas negative weighted mean differences reflect higher regulatory activity the former (IPSCs).

***.Summary.xls files:** pairwise comparison of read density for TF-specific TFBS (FTPs) is computed between the indicated time points. Representative TFs are also displayed in the volcano plots (Fig. 5). Weighted_meanDifference: indicates the difference in accessibility between time points, hence differential TF activity between time points. TFBS is the number of predicted TF binding sites (FTPs) that overlap peak regions called at both time points and upon which the weighted mean difference between time points is based on. Briefly, if higher TF binding activity results in higher target gene expression output, the TF will be classified as activator. If higher TF binding activity results, instead, in lower target gene expression, then the TF will be classified as repressor [16].
For each time point, real (TF-specific FTPs) and background distribution (peaks regardless of TFBS) are calculated as the weighted mean across all CG bins. The difference is computed on the means of the log2 fold-change values, weighted for CG bins, from foreground and background. weighted_CD: the Cohen's d measure of the effect size, weighted by CG bin. TFBS: the number of predicted TF binding sites for the particular TF that overlap with the peaks and that the analysis was based on. *p*-val adjusted: *p*-value assesses the significance of the obtained weighted_meanDifference adjusted by multiple Hp correction (using Benjamini-Hochberg). The resulting *p*-value is based on the weighted_Tstat and variance. median.cor.tfs: The median value for the RNA-Seq-ATAC-Seq correlations from the foreground (i.e., peaks with a predicted TFBS for the particular TF). Classification: A TF is either classified as activator, undetermined, repressor, or not expressed. Classification across stringencies (q-val 0.1 to 0.001); classification_distr_rawP: The raw *p*-value of the one-sided Wilcoxon rank sum test for step 2. For TFs that were classified as either repressor or activator after step 1 but for which the raw *p* value of the Wilcoxon rank sum test was not significant, their classification was changed to undetermined, removing TF classifications with weak support. Classification final (q-val 0.1 to 0.001): TF classifications after step 2 (final, as in the Volcano plot).

***.Peak_distribution.xls:** permutation; TF; Pos_l2FC; mean_l2FC; median_l2FC; mode_l2FC; sd_l2FC; *p*-val_raw; skewness_l2FC; t-stat; TFBSnum; adj_*p*-val; log2 fold change distribution across all TFBS and differences between all TFBS and the peaks [16]. TF-specific results for the differential accessibility analysis and have the following columns: - TF: name of the TF - permutation: The number of the permutation. Pos_log2FC, Mean_log2FC, Median_log2FC, sd_log2FC, Mode_log2FC, skewness_log2FC: fraction of

positive values, mean, median, standard deviation, mode value and Bickel's measure of skewness of the log2 fold change distribution across all TFBS. *P*-value_raw and *p*-value_adj: raw and adjusted (fdr, i.e. Bonferroni-Hochberg) *p*-value of the t-test, T_statistic: the value of the T statistic from the t-test -TFBS_num: number of TFBS. Diff_mean, Diff_median, Diff_mode, Diff_skew: difference of the mean, median, mode, and skewness between the log2 fold-change distribution across all TFBS and the peaks, respectively.

Supplementary Materials: The following supporting information can be downloaded at: www.mdpi.com/xxx/s1, Supplementary Figures S1-S11, Excel tables, and peak files.
